# Kratom use disorder and unfolded protein response: Evaluating their relationship in a case control study

**DOI:** 10.1371/journal.pone.0287466

**Published:** 2023-06-23

**Authors:** Bin Yang, Mei Lan Tan, Ruiling Zhang, Darshan Singh, Mohammad Farris Iman Leong Bin Abdullah

**Affiliations:** 1 Department of Community Health, Advanced Medical and Dental Institute, Universiti Sains Malaysia, Kepala Batas, Pulau Pinang, Malaysia; 2 School of Pharmaceutical Sciences, Universiti Sains Malaysia, Gelugor, Pulau Pinang, Malaysia; 3 Second Affiliated Hospital, Xinxiang Medical University, Xinxiang, Henan, China; 4 Centre for Drug Research, Universiti Sains Malaysia, Gelugor, Pulau Pinang, Malaysia; University of Sri Jayewardenepura, SRI LANKA

## Abstract

**Background and aims:**

Kratom (*Mitragyna speciosa* Korth.) is widely use worldwide despite its addictive potential. Although psychostimulant use has been linked to occurrence of endoplasmic reticulum (ER) stress, data is lacking on how regular kratom use affects ER stress. This case-control study first determined differences in ER stress sensor protein expression (BiP, sXBP1, ATF4, CHOP, JNK, and p-JNK) between regular kratom users and healthy controls. Second, it evaluated the association between kratom use characteristics, targeted ER stress sensor protein expression, and “kratom use disorder” diagnosed with Diagnostic and Statistical Manual for Mental Disorders 5^th^ Edition (DSM-5) among regular kratom users.

**Methods:**

In total, 60 regular kratom users and 50 healthy control-group participants were recruited and administered a sociodemographic and clinical characteristics questionnaire. While participants who used kratom were also administered a kratom use characteristics questionnaire. Blood samples were collected from all participants, and targeted ER stress sensor protein expression was determined via Western blot analysis.

**Results:**

The study’s findings revealed first that kratom users registered significantly higher protein expression in all targeted ER stress sensors compared to the control group. Second, higher protein expression of CHOP (*B* = 5.061, standard error [SE] = 2.547, Wald = 3.948, adjusted odds ratio [AOR] = 5.382, 95% confidence interval [CI] = 1.071 to 9.656, p = 0.047) and p-JNK (*B* = 5.795, SE = 2.635, Wald = 4.544, AOR = 17.025, 95% CI = 1.395 to 24.123, p = 0.017) increased the odds of kratom use disorder occurrence. Kratom use characteristics and other ER stress sensor protein expression were not associated with kratom use disorder.

**Conclusion:**

Regular kratom use may induce protracted ER stress, leading to the decompensation of the unfolded protein response to maintain ER homeostasis. This effect may be linked to kratom use disorder occurrence.

## Introduction

Kratom (*Mitragyna speciosa* Korth.) is traditionally used in remedies for various illnesses in Southeast Asia. Currently, it is widely used around the world to improve mood and anxiety, enhance endurance, manage opioid dependence as a self-prescribed replacement, and serve as an analgesic for chronic pain [1]. Despite its therapeutic potential, kratom’s capacity to induce withdrawal symptoms when users abstain, as well as the risk of kratom dependence due to its opioid-like effects, has been a major drawback to its potential as a therapeutic agent, particularly for users who consumed an average quantity higher than three glasses of kratom juice daily [2, 3]. Singh et al. (2019) also reported, in a cross-sectional survey of regular kratom users, that 99% of users had kratom-related substance use disorder; 95% presented with withdrawal symptoms, 87% with tolerance, and 93% with cravings while abstaining from kratom use [3]. A recent survey in the US also pointed out the proportion of current and former kratom users with a diagnosis of “kratom use disorder” by referring to Diagnosis and Statistical Manual for Mental Disorders 5^th^ Edition (DSM-5) substance use disorder for kratom was at 47.3% [4].

The mechanism underlying occurrence of kratom dependence is still unclear, but evidence from animal study began to emerge indicating the mitragynine, the commonest psychoactive alkaloid constituent of kratom may reduced gamma-aminobutyric acid (GABA) release and inversely enhanced dopamine secretion which may contribute to kratom dependence [5]. In essence, kratom exhibited psychostimulant effects at lower dosage [6] and hence, may share similar properties as psychostimulant drugs, such as cocaine and methamphetamine. Administration of cocaine and methamphetamine is linked to activation of endoplasmic reticulum (ER) stress. ER stress occurs when the protein folding in the ER lumen is perturbed, leading to the accumulation of misfolded and unfolded protein in the ER. An unfolded protein response (UPR) responds to ER stress, aiming to counteract this stress by reducing unfolded or misfolded protein in order to maintain ER homeostasis. However, if UPR cannot counteract ER stress, persistent ER stress will ultimately lead to cell apoptosis [7]. Several mental illnesses are associated with the occurrence of ER stress, such as depression, bipolar mood disorder, posttraumatic stress disorder, and psychostimulant dependence [8, 9]. In the context of psychostimulant use, acute administration of cocaine trigger the expression of ER stress sensors, such as BiP, PERK, and IRE1α in the dorsal striatum related to stimulation of the D1 dopaminergic and the N-methyl-D-aspartate (NMDA) receptors. Similarly, acute administration of methamphetamine also induced upregulation of BiP and CHOP in the dorsal striatum and stimulate the increase expression of BiP and ATF4 in the ventral tegmental area and substantia nigra [10]. However, data is lacking on how kratom intake which also exhibits psychostimulant effect [6], would affect the ER stress and UPR and how the alteration in ER stress sensors are related to the severity of kratom dependence.

Hence, the current study filled this research gap by investigating how chronic or regular kratom use affected ER stress and UPR. First, this investigation compared ER stress sensor protein expression (BiP, sXBP1, ATF4, CHOP, JNK, and p-JNK) between regular kratom users and a control group of healthy, non-drug-using participants (Objective 1). Second, it determined the association between kratom use characteristics, targeted ER stress sensor protein expression and kratom use disorder diagnosed using DSM-5 [11] among regular kratom users (Objective 2).

## Materials and methods

### Study design and participants

This case-control study was conducted from January 2019 to December 2020. Subject recruitment and data collection commenced from January 2019 to January 2020. It was approved by the Human Research Ethics Committee of Universiti Sains Malaysia (code: USM/JEPeM/18100568), and it abides by the Declaration of Helsinki (1975) and its revisions. The sample size for Objective 1 was estimated using the G*Power 3.1.9.7 sample size calculator to account for the difference between two independent means. The Type I error was 0.05, the power was 0.8, the allocation ratio was 1:1, and the effect size was 0.6 (regarding a study that compared ER stress sensor mRNA expression between depressed and control subjects [12]). The estimated sample size was 98 subjects with 49 subjects per group (including a 10% dropout rate). The sample size for Objective 2 was estimated using the same sample size calculator to account for multiple logistic regression. The Type I error was 0.05, the power was 0.8, and the effect size was 0.40 [13]. Objective 2’s estimated sample size was 52 regular kratom users (including a 20% dropout rate).

**Fig 1:**
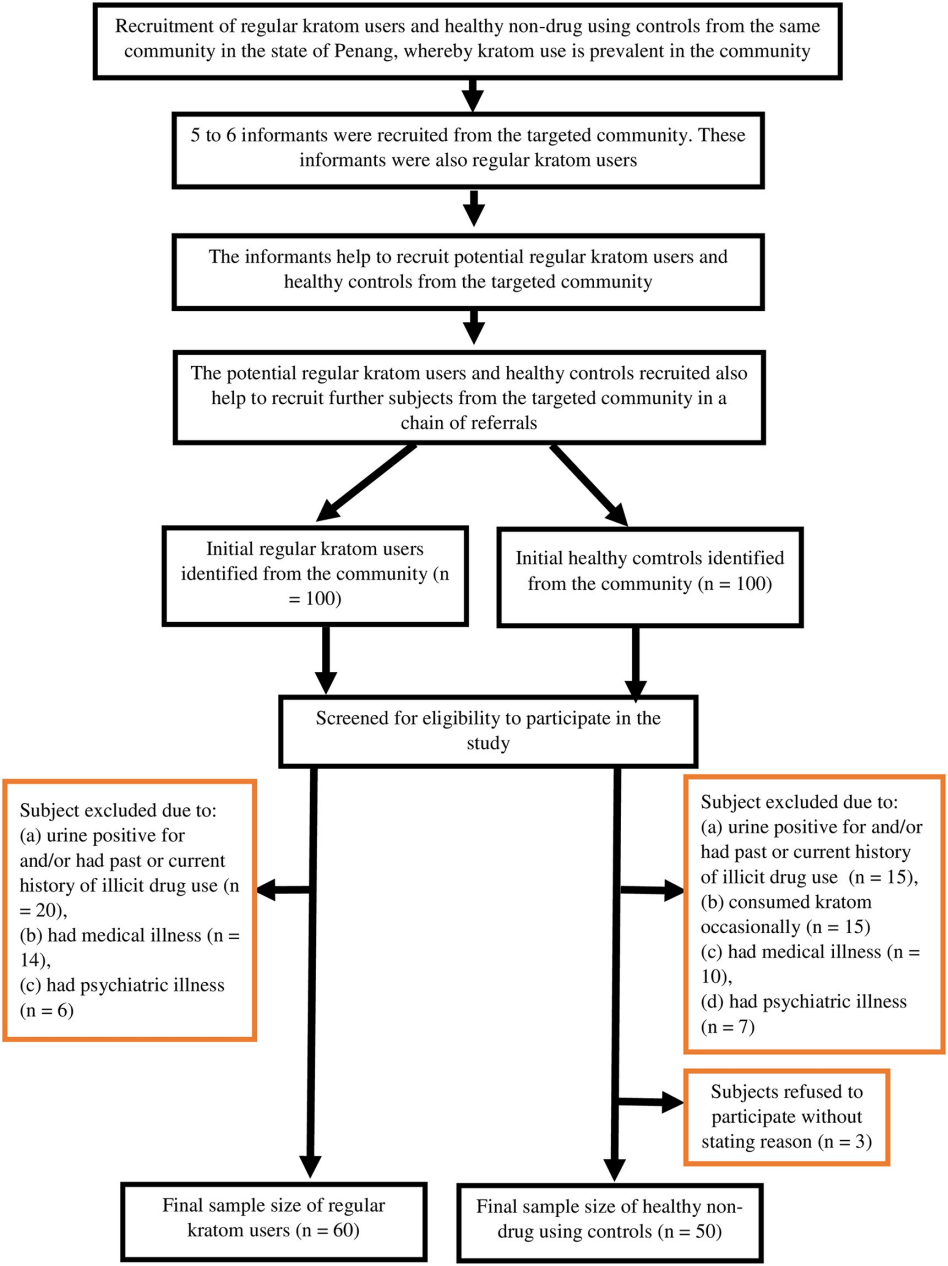
Flow chart of the chronology of events during subject recruitment of this study.

This study’s participants were recruited using exponential discriminative snowball sampling. Regular kratom users and healthy, non-drug-using control-group participants were recruited from the same targeted community in the State of Penang in Malaysia. Fig 1 summarizes the chronology of this participant recruitment. Initially, five to six informants who regularly used kratom were recruited from the targeted community to assist the research team in recruiting participants. Then, the informants recruited potential regular kratom users and healthy control-group participants, who referred additional prospective participants to the study. However, subjects with varying demographic and social characteristics (age, monthly household income, education attainment, occupation, family support, family ties among subjects, and social circle) were selected for eligibility criteria screening to increase the heterogeneity of the subjects in order to reduce selection bias. None of the kratom users and healthy controls were family members. Next, the research team screened the prospective participants to determine their eligibility for the study. The inclusion criteria for regular kratom users included: (1) a daily kratom consumption history of 12 consecutive months (we defined regular kratom use as having history of daily consumption of kratom for 12 consecutive months adapted from other studies of kratom users in Malaysia [2, 3], and from DSM-5 diagnostic criterion for substance use disorder), (2) an age of 18 to 60 years old, and (3) the ability to speak and write in the Malay language. The exclusion criteria for kratom users were: (1) a history of past or current psychiatric illness, (2) a history of past or current medical illness, (3) a history of past or current illicit drug or alcohol use, and (4) a past or current history of regularly using medications that could alter ER stress. The eligibility criteria for the healthy control group were similar to those for kratom users, except that this group’s exclusion criteria included a past or current history of kratom consumption.

Initially, 100 potential kratom users and 100 potential control subjects were identified by snowball sampling. After being screened for the study’s eligibility criteria, 40 kratom users were excluded due to positive urine results for illicit drug consumption and past or current illicit drug use, medical illness, or psychiatric illness. Similarly, 47 prospective control-group participants were excluded due to a history of occasional kratom consumption, positive urine results for an illicit drug, and past or current illicit drug use, medical illness, or psychiatric illness. Additionally, three prospective control-group participants were excluded because they refused to participate in the study for no stated reason. Hence, in total, 60 regular kratom users and 50 healthy, non-drug-using control-group members participated in the study (Fig 1). Then, the participants signed informed consent forms before enrolling in the study. Participants’ personal identifiable information will not be elicited and they are assured of their participation anonymity.

### Screening for medical and psychiatric illness, illicit drug use and regular medication use

(1) Screening for past and current medical illness:

Screening for past and current medical illness in all the participants were performed by the medical doctor in the research team. Detail history on medical illness which could induced psychiatric symptoms and ER stress were elicited from all potential subjects (kratom users and healthy controls), such as acute myocardial infarction, congestive cardiac failure, ischemic heart disease, bronchial asthma, chronic obstructive airways disease, cancer, cerebrovascular accident, myasthenia gravis, multiple sclerosis, epilepsy, systemic lupus erythematosus, osteoarthritis, rheumatoid arthritis, thyrotoxicosis, hypothyroidism, Cushing’s syndrome, adrenal insufficiency, phaeochromocytoma, chronic and end-stage renal failure, acute glomerulonephritis, nephrotic syndrome, diabetic complications, hypertensive complications, gout, hepatitis, and liver cirrhosis. General and systemic examination were performed to assess the respiratory system, cardiovascular system, central and peripheral nervous system, endocrine system, joints, gastrointestinal system, and genitourinary system. In addition, blood investigations were also carried out, such as full blood count, renal profile and serum electrolytes, liver function test, thyroid function test, fasting blood lipid and fasting blood sugar. Those who had any past and current history of any medical illnesses that were screened were excluded from the study. Hence, all the participants in the study (regular kratom users and healthy controls) had unremarkable findings in history, examination and also blood investigations. The blood investigation findings are summarized in S1 Table. Mann-Whitney U test revealed that there was no difference in all the parameters of the blood investigations between kratom users and healthy controls except total white blood cells (WBC) and hematocrit, whereby the total WBC of kratom users was significantly higher than that of healthy controls (p = 0.043), whilst hematocrit of healthy controls was significantly higher than kratom users (p = 0.043). However, both total WBC and hematocrit of kratom users and healthy controls were still within normal range.

(2) Screening for past and current psychiatric illness:

Screening for past and current history of psychiatric illness were carried out by the psychiatrist in the research team. All potential subjects (kratom users and healthy controls) were screened with Diagnostic and Statistical Manual for Mental Disorders 5th Edition (DSM-5) diagnostic criteria and Mini International Neuropsychiatric Interview [14] for mental illnesses, such as:

- Depressive disorders (major depressive disorder, persistent depressive disorder, premenstrual dysphoric disorder, depressive disorder due to substance or medication use, and depressive disorder due to another medical condition),
- Anxiety disorders (generalized anxiety disorder, panic disorder, social anxiety disorder, specific anxiety disorder, and agoraphobia, substance/medication induced anxiety disorder, and anxiety disorder due to another medical condition),
- Psychotic disorders (schizophrenia, schizophreniform disorder, schizoaffective disorder, brief psychotic disorder, delusional disorder, psychotic disorder due to another medical condition, and substance/medication induced psychotic disorder),
- Bipolar I disorder, bipolar II disorder, cyclothymic disorder, substance/medication induced bipolar and related disorder, and bipolar and related disorder due to another medical condition,
- Obsessive-compulsive related disorders (obsessive compulsive disorder, hoarding disorder, and body dysmorphic disorder),
- Trauma-related disorders (acute stress disorder, posttraumatic stress disorder, and adjustment disorder),
- Others (autism spectrum disorder, adult attention deficit hyperactive disorder, major neurocognitive disorder, personality disorders)

Those who had any past and current history of any psychiatric illnesses that were screened were excluded from the study. Hence, all the participants in the study (regular kratom users and healthy controls) had unremarkable findings in history and mental state examination.

(3) Screening for past and current history of illicit drug and alcohol use:

Screening for past and current history of illicit drug use were carried out by the psychiatrist in the research team. Detailed drug use history were elicited among all the potential subjects for past and current use of illicit drugs or substance, such as alcohol, opioids (heroin, morphine, methadone, pethidine, and buprenorphine), amphetamine-type-stimulant (methamphetamine, 3,4-methylenedioxymethamphetamine, phentermine, and methylphenidate), ketamine, cannabis, cocaine, phencyclidine, hallucinogens, benzodiazepine (diazepam, clonazepam, lorazepam, alprazolam, midazolam, and nimetazepam), and volatile substances (glue, petrol, paints). In addition to detailed history from subjects, history from close family members and neighbours were also elicited to consolidate history from subjects. Rapid urine test (from Fast Screen Multi Drugs Dipcard, Reszon Diagnostics International Sdn. Bhd., Subang Jaya, Malaysia) was also carried out to screen for illicit drugs, such as opioid, methamphetamine, ketamine, cannabis, PCP, and benzodiazepine. Those who had any past and current history of any illicit drug and alcohol use that were screened were excluded from the study. Hence, all the participants in the study (regular kratom users and healthy controls) were all cleared of any past and current history of illicit drug and alcohol use.

(4)Screening for past and current history of regular medication use that could alter ER stress:

Screening for past and current history of regular medication use were carried out by the medical doctor in the research team. Detailed medication use history were elicited among all the potential subjects, to search for history of regular medication use which could alter ER stress, such as arsenic trioxide, bleomycin, bortezomib, cisplatin, clozapine, olanzapine, cyclosporin, diclofenac, indomethacin, efavirenz, protease inhibitors, thapsigargin, tunicamycin, zidovudine, and sertraline [15]. Those who had any past and current history of any regular medication use that could alter ER stress were excluded from the study. Hence, all the participants in the study (regular kratom users and healthy controls) were all cleared of any past and current history of regular medication use that could alter ER stress.

### Measures

#### Outcome variable

The outcome variable was diagnosis of substance use disorder related to kratom use or “kratom use disorder” among kratom users according to the diagnostic criteria of DSM-5 (the diagnostic criteria are as follow: (1) taking kratom in larger amounts and for longer than intended; (2) wanting to cut down or quit kratom but not being able to do it; (3) spending a lot of time obtaining kratom, use it or recover from its effect; (4) craving or a strong desire to use kratom, (5) repeatedly unable to carry out major obligations at work, school, or home due to kratom use; (6) continued kratom use despite persistent or recurring social or interpersonal problems caused or made worse by kratom use, (7) stopping or reducing important social, occupational, or recreational activities due to kratom use, (8) recurrent use of kratom in physically hazardous situations, (9) consistent use of kratom despite acknowledgment of persistent or recurrent physical or psychological difficulties from using kratom, (10) tolerance to kratom which is a need for markedly increased amounts to achieve desired effect or markedly diminished effect with continued use of the same amount of kratom, and (11) withdrawal from kratom when abstain from kratom use. The presence of at least two of the above diagnostic criteria for 12 consecutive months fulfilled a diagnosis of kratom use disorder, in which assessment of participants was performed by the psychiatrist in the research team using the DSM-5 diagnostic criteria. This assessment classified regular kratom users into “those with kratom use disorder” or “those without kratom use disorder”.

#### Explanatory variables

*(1) Targeted ER stress sensor protein expression*. Biomarkers have been applied in clinical practice for several decades, which can objectively measure normal or pathological state of the organism and be used as objective indicators to evaluate the effects of therapeutic interventions [16]. Many biomarkers have been found in body fluids, including blood, urine, stool, saliva, cerebrospinal fluid, semen, and cervico-vaginal fluid, and Blood is the most common source of biomarkers [17]. Current studies have shown that PBMCs from blood have become potential biomarkers for metabolic pathways and pathogenesis of many diseases [18]. PBMCs is the most versatile tool as compared to using samples obtained from other parts of the body (eg brain tissues or liver tissues), which are invasive methods and certainly not practical. Hence, PBMCs would be a potentially good sample to study its effects. PBMCs serve as a “window” to the central nervous system. PBMCs are one of the main antigen presenting cells in the peripheral blood and has been reported to play crucial role in communicating with other cells. There are approximately 500 mL of cerebrospinal fluid (CSF) absorb into the bloodstream daily in which psychiatric conditions which lead to neurodegeneration such as substance use disorder and Alzheimer’s disease may induce blood brain barrier dysfunction and protein exchange between the body fluid (peripheral blood and CSF). Hence, ER stress that occurred in the brain cells may be represented in the PBMCs [19–21]. However, it remains indistinct whether PBMCs partakes the metabolic process of kratom in the body and the detailed mechanism needs to be further explored.

In this study, blood samples were collected from all participants (kratom users and healthy controls). The targeted ER stress sensor protein which were extracted from the peripheral blood mononuclear cells and quantified included binding immunoglobulin protein (BiP), spliced X-box-binding protein 1 (sXBP1), activating transcription factor 4 (ATF4), c-Jun N-terminal kinase (JNK), phosphorylated c-Jun N-terminal kinase (p-JNK), and CCAAT-enhancer-binding protein homologous protein (CHOP). The ER stress sensor protein extraction and quantification was determined using Western blot.

The procedures for Western blot to determine the targeted ER stress protein expression are as follow:

EDTA K2 tubes were used to collect peripheral blood from participants (a 7.5 mL blood sample from each participant). Ficoll-Paque Plus (GE Healthcare, United States) was used to isolate peripheral blood mononuclear cells according to the manufacturer’s instructions. RIPA Lysis Buffer (Beyotime, China), supplemented with Protease and a phosphatase inhibitor cocktail (Beyotime, China), was used to isolate total proteins. Then, the proteins were electrophoretically transferred to the polyvinylidene difluoride membrane after they were separated by sodium dodecyl sulfate–polyacrylamide gel electrophoresis (SDS-PAGE). After blocking, the membranes were incubated with the rabbit sXBP1 monoclonal antibody (1:1 000, CST, United States), rabbit BiP-specific monoclonal antibody (1:1 000, CST, United States), rabbit JNK-specific monoclonal antibody (1: 1 000, CST, United States), rabbit p-JNK-specific monoclonal antibody (1:1 000, CST, United States), rabbit ATF4-specific monoclonal antibody (1:1 000, Abcam, United Kingdom), mouse CHOP-specific monoclonal antibody (1:1 000, CST, United States), and mouse β-actin monoclonal antibody (1: 1 000, Abcam, United Kingdom) overnight at 4°C. The next day, the membranes were incubated with horseradish peroxidase-conjugated goat anti-rabbit/mouse IgG (1:1 000, Beyotime, China). VersaDoc^™^ MP Imaging Systems (Bio-Rad Laboratories, United States) were used to expose and collect images. Grayscale analysis of the results was performed using Image J. The proteins’ relative expression was computed based on the signals of the corresponding β-actin band. The raw images of the Western blot analysis of the targeted ER stress sensors in this study are illustrated in S1 Fig.

*(2) Kratom use characteristics*. The kratom use characteristics recorded include kratom users’ average frequency of daily kratom use, average quantity of daily kratom use, and duration of kratom use. The questions and response options for each kratom use characteristic are depicted in S1 Appendix. The average frequency of daily kratom use was recorded based on the question “On average, how frequent do you drink kratom juice on a daily basis?”. The response options to the question were coded as “1 to 3 times per day “or “more than 3 times per day”. The average quantity of daily kratom use was assessed based on the following question “On average, how many glasses of kratom juice do you drink on a daily basis?”. The response options to the question were coded as “1 to 3 glasses per day (whereby 1 glass is equivalent to approximately 300 ml of kratom juice)” or “more than 3 glasses per day (whereby 1 glass is equivalent to approximately 300 ml of kratom juice)”. The duration of kratom use was reported based on the question “How long have you been consuming kratom?”. The response options to the question were coded as “For the past 1 to 6 years” or “more than 6 years”.

#### Sociodemographic and clinical characteristics

All participants (kratom users and healthy controls) were administered a sociodemographic and clinical characteristics questionnaire. The sociodemographic characteristics that were recorded for the participants included age, ethnicity, employment status, marital status, education status, and monthly income. The responses for age were coded as “less than 40 years old” or “40 years old and above”. The responses for ethnicity were coded into “Malay” or “non-Malay. The responses for employment status were coded as “employed” or “non-employed/student”. The responses for education level were coded as “up to secondary education” or “tertiary education”. The responses for marital status were coded as “married” or “single/divorce/widower”. Finally, the responses for monthly income were coded as “Malaysian Ringgit 1000 and below” or “above Malaysian Ringgit 1000”.

The clinical characteristics that were documented included a history of cigarette smoking, body mass index (BMI; measured as weight [in kg] per square unit of height [in m2]), blood pressure (measured as the average of two repeated readings in mmHg), and pulse rate (measured in beats per minute). The response options for history of cigarette smoking were coded as “non-smoker”, “those who smoke 10 sticks per day or below” or “those who smoke more than 10 sticks per day”. The response options for blood pressure were coded as “normal which was < 130/80 mmHg” or “high which was ≥ 130/80 mmHg” (none of the participants had low blood pressure which would be < 90/60 mmHg). While the response options for pulse rate were coded as “normal which was < 100 beats/minute” or “high which was ≥ 100 beats/minute (none of the respondents had low pulse rate which would be < 60 beats/minute). The response options for each demographic and clinical characteristic are presented in S2 Appendix.

### Statistical analysis

All data analysis was performed with Statistical Package for Social Sciences, Version 26 (SPSS 26; Chicago, IL, United States). Descriptive statistics for sociodemographic and clinical characteristics, kratom use characteristics, KDS scores, and targeted ER stress sensors’ protein expression (BiP, sXBP1, ATF4, CHOP, JNK, and p-JNK) were computed. All categorical data were presented as frequencies and percentages. Meanwhile, all continuous data were computed as medians and interquartile ranges (IQR) since the data were not normally distributed, as the Shapiro–Wilk test had indicated. There was no missing data.

To achieve this study’s first objective, differences in the targeted ER stress sensors’ protein expression between regular kratom users and healthy control-group participants were evaluated with the Mann–Whitney U test. Sensitivity analysis was assured by intensive screening of participants (kratom users and controls) for medical illness, psychiatric illness and regular intake of medication and substances to exclude subjects with these conditions, which could affect ER stress. To achieve Objective 2, the associations between the kratom use characteristics, targeted ER stress sensor protein expression (independent variables), and kratom use disorder (dependent variable) were determined using multiple logistic regression model after controlling for confounding factors such as age, blood pressure, and cigarette smoking [22–24]. For kratom use disorder, absence of kratom use disorder was assigned a dummy code of 0 (reference) and presence of kratom use disorder was allocated a dummy code of 1. All statistical significance was set to p < 0.05 and two-sided.

## Results

### Participants

All participants’ sociodemographic and clinical characteristics are summarized in S2 Table. While the kratom use characteristics among the regular kratom users who participated in this study are presented in S3 Table. The prevalence of kratom use disorder among the regular kratom users in this study was 41.7% (n = 25).

### Comparing ER stress sensors’ protein expression between regular kratom users and healthy control-group members

**Fig 2:**
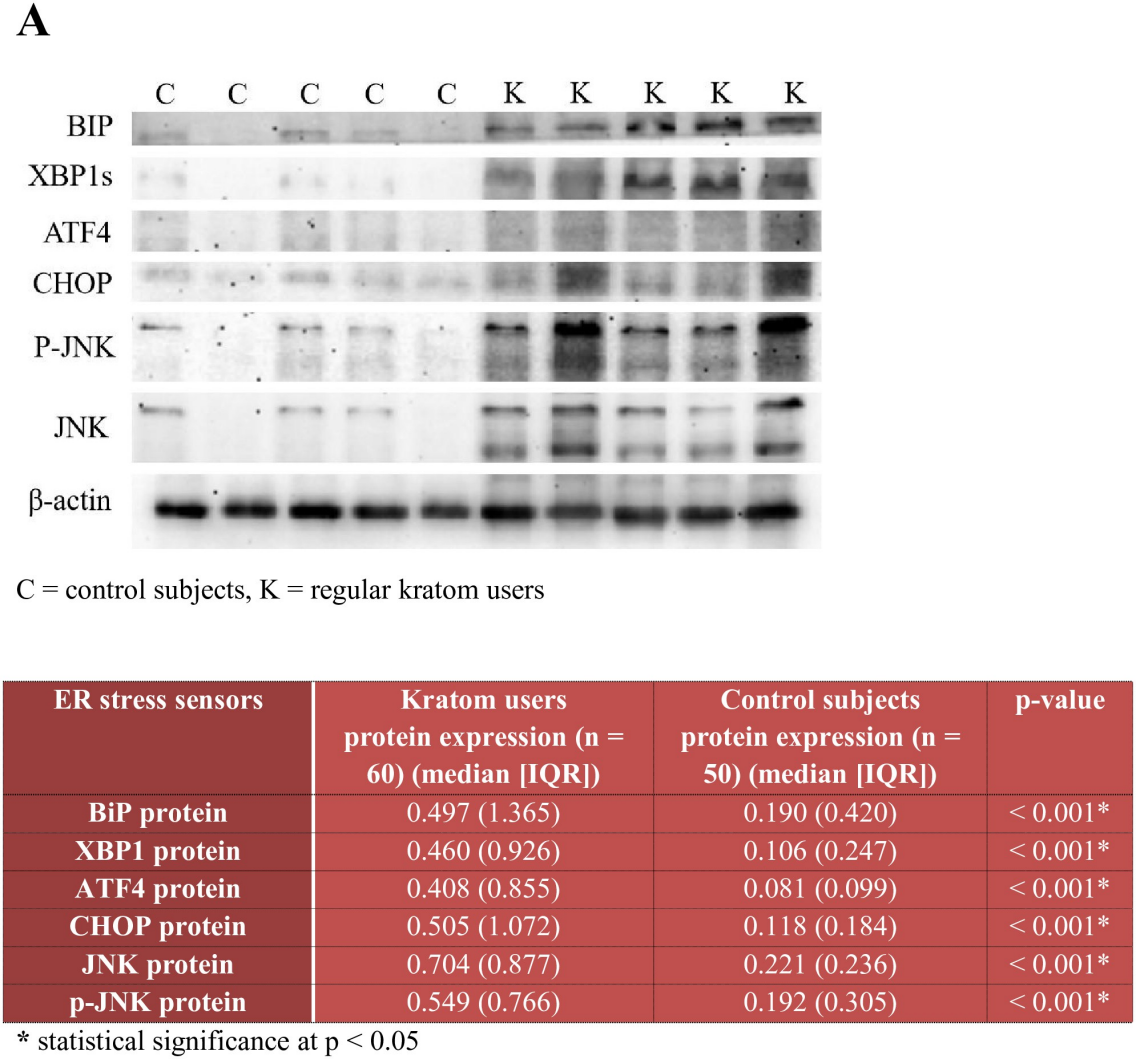
The differences in targeted ER stress sensor protein expression between regular kratom users and healthy non-drug using controls computed using Mann Whitney U test. From the findings above, the median ER stress sensors protein expression (BiP, sXBP1, ATF4, CHOP, JNK and p-JNK) of the regular kratom users were all significantly higher than that of the healthy controls (all p values < 0.001). A = Photographic representation of the Western blot band of targeted ER stress sensors relative to β-actin band. Abbreviations: BiP = binding immunoglobulin protein, sXBP1 = spliced X-box-binding protein 1, ATF4 = activating transcription factor 4, JNK = c-Jun N-terminal kinase, p-JNK = phosphorylated c-Jun N-terminal kinase, CHOP = CCAAT-enhancer-binding protein homologous protein.

Differences in the targeted ER stress sensor protein expression between the regular kratom users and healthy control-group members are depicted in Fig 2. The regular kratom users registered significantly higher BiP, sXBP1, ATF4, CHOP, JNK, and p-JNK protein expression than the control group. Higher protein expression was recorded for JNK, p-JNK, and CHOP than for BiP, sXBP1, and ATF4 among the kratom users (Fig 2).

### The association between kratom use characteristics, targeted ER stress sensor protein expression, and kratom use disorder among the regular kratom users

The association between kratom use characteristics, targeted ER stress sensor protein expression (independent variables), and kratom use disorder (dependent variable) after controlling for confounding factors (such as age, cigarette smoking, and blood pressure) among the regular kratom users are summarized in Table 1. From the multiple logistic regression model, two of the targeted ER stress sensors protein expression contributed to higher occurrence of kratom use disorder. Higher protein expression of CHOP was associated with increase odds of kratom use disorder by 5.382 folds (*B* = 5.061, SE = 2.547, Wald = 3.948, AOR = 5.382, 95% CI = 1.071 to 9.656, p = 0.047). Similarly, higher protein expression of p-JNK was associated with increase odds of kratom use disorder by 17.025 folds (*B* = 5.795, SE = 2.635, Wald = 4.544, AOR = 17.025, 95% CI = 1.395 to 24.123, p = 0.017). None of the kratom use characteristics were associated with the odds of occurrence of kratom use disorder.

**Table 1:** The association between the kratom use characteristics, targeted ER stress sensor protein expression (BiP, sXBP1, ATF4, CHOP, JNK, and p-JNK) and substance use disorder related to kratom use among regular kratom users after controlling for confounding factors (such as age, cigarette smoking, and blood pressure) in a multiple logistic regression model.

Variables	*B*	SE	Wald	AOR (95% CI)	p-value
**Average daily frequency of kratom use**:					
1 to 3 times/day	Reference				
> 3 times/day	-4.105	2.481	2.737	0.016 (0.001 to 1.239)	0.098
**Average daily quantity of kratom use**:					
1 to 3 glasses of kratom juice/day	Reference				
> 3 glasses of kratomjuice/day	0.753	1.987	0.144	2.124 (0.043 to 54.256)	0.705
**Duration of kratom use**:					
1 to 6 years	Reference				
> 6 years	-3.601	1.846	3.807	0.022 (0.001 to 1.239)	0.063
**Age**:					
< 40 years old	Reference				
≥ 40 years old	-2.259	2.641	0.732	0.104 (0.001 to 9.490)	0.392
**Blood pressure**:					
Normal (< 130/80 mmHg)	Reference				
High (≥ 130/80mmHg)	-1.253	2.281	0.302	0.286 (0.003 to 12.981)	0.583
**Cigarette smoking**:					
≤ 10 stick/day	Reference				
> 10 sticks/day	-0.026	0.562	0.002	0.974 (0.323 to 2.933)	0.963
**BiP protein expression**	0.501	1.587	0.100	1.651 (0.074 to 18.063)	0.752
**sXBP1 protein expression**	-5.076	4.002	1.609	0.006 (0.000 to 8.913)	0.205
**ATF4 protein expression**	5.650	3.070	3.573	2.897 (0.293 to 5.987)	0.120
**CHOP protein expression**	5.061	2.547	3.948	5.382 (1.071 to 9.656)	0.047*
**JNK protein expression**	-0.868	1.782	0.237	0.420 (0.013 to 13.810)	0.626
**p-JNK protein expression**	5.795	2.635	4.544	17.025 (1.395 to 24.123)	0.017*

* statistical significance at p < 0.05,

SE = standard error, AOR = adjusted odds ratio, 95% CI = 95% confidence interval of odds ratio, multiple logistic regression model: Hosmer and Lemeshow test (p = 0.998), Nagelkerke R^2^ = 0.858

## Discussion

**Fig 3:**
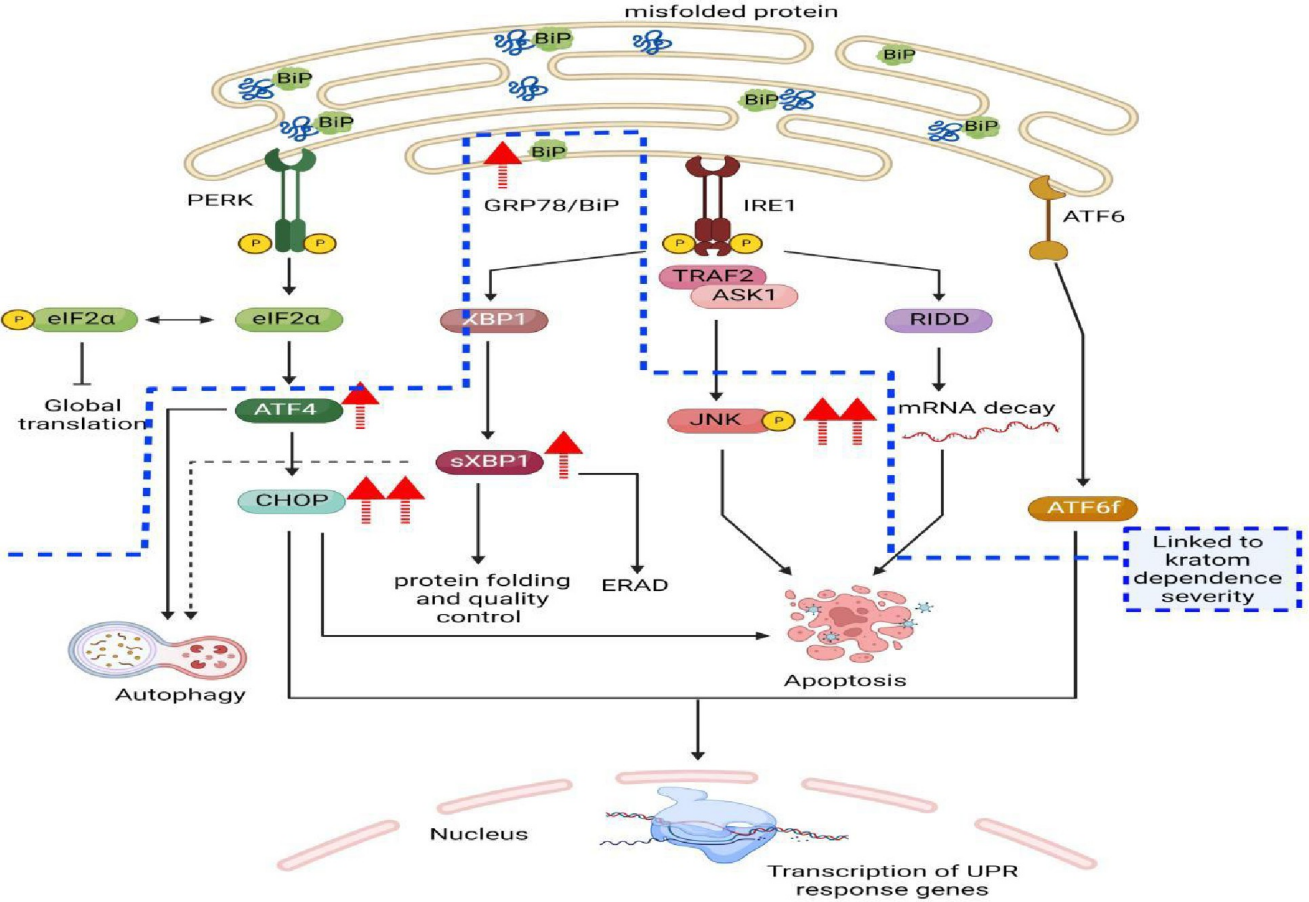
Summary of the findings of this study. The targeted ER stress sensor protein expressions were determined by Western blotting, which were presented as relative protein expression or fold change by comparing with the corresponding β-actin band. There were higher BiP protein expression in regular kratom users compared with healthy controls indicating greater degree of ER stress and UPR occurred upon chronic kratom consumption. The higher degree of protein expression of sXBP1, ATF4, CHOP, JNK, and p-JNK may demonstrate activation of the PERK-eIF2α-ATF4-CHOP, the IRE1α-sXBP1-CHOP, and the IRE1α-JNK-p-JNK pathways of the ER stress as well as persistence of ER stress which may have decompensated the UPR capacity to restore ER homeostasis. In addition, elevation of protein expression of CHOP and p-JNK, indicating a link between protracted ER stress, UPR and higher odds of occurrence of “kratom use disorder”. Hence, activation of the PERK-eIF2α-ATF4-CHOP, the IRE1α-XBP1-CHOP, and the IRE1α-JNK-p-JNK pathways of the ER stress may be associated with occurrence of “kratom use disorder” (publication and licensing rights for Fig 3 is illustrated in S3 Appendix). Abbreviations: ER = endoplasmic reticulum, UPR = unfolded protein reaction, ERAD = endoplasmic reticulum-associated degradation, PERK = protein kinase RNA-like ER kinase, IRE1α = inositol-requiring transmembrane kinase/endoribonuclease 1α, ATF6 = activating transcription factor 6, eIF2α = eukaryotic translation initiation factor 2A, TRAF2 = Tumor necrosis factor receptor-associated factor 2, ASK1 = Apoptosis signal-regulating kinase 1, and RIDD = regulated IRE1-dependent decay.

This study investigated how regular kratom use affected kratom users’ ER stress and UPR, as well as how alterations to ER stress and UPR was associated with the odds of occurrence of kratom use disorder. Our findings revealed that the relative protein expression of all the targeted ER stress sensors was higher among kratom users than healthy control-group participants. Additionally, the relative protein expression of CHOP, JNK, and p-JNK was higher than that of BiP, sXBP1, and ATF4 among kratom users (Fig 2). Finally, after controlling for kratom use characteristics in the multiple logistic regression model, higher CHOP and p-JNK protein expression were associated with higher odds of occurrence of kratom use disorder. Hence, we summarized the findings of this study in Fig 3.

ER stress occurs when unfolded and misfolded protein accumulates in the ER lumen. Initially, UPR is activated to counteract this ER stress by: inhibiting the translation of protein; upregulating the ER chaperone genes to help fold, assemble, and modify protein in the ER lumen; and increasing the degradation of unfolded and misfolded protein via ER-associated protein degradation (ERAD) [25, 26]. In the absence of ER stress, BiP or GRP78 keeps the three ER transmembrane proteins—PERK, IRE1α, and ATF6 (primarily responsible for regulating UPR)—in an inactive state. When ER stress occurs, BiP dissociates from the three transmembrane proteins which lead to activation of PERK, IRE1α, and ATF6, and UPR is initiated. BiP is also involved as an ER chaperone to help with protein folding and shaping, as well as protein degradation in the UPR [27]. Hence, the higher BiP protein expression among kratom users compared to healthy control-group participants in our study indicates that a greater degree of ER stress and UPR occurs with chronic kratom consumption (Figs 2 and 3).

First, in the IRE1α regulated pathway, after BiP dissociates from IRE1α, the latter undergoes dimerization and autophosphorylation to become activated, which then initiates the transcription of XBP1 mRNA. This activation also regulates kinase and endonucleases to cut a portion of XBP1 intron (of 26 nucleotides), forming the active form of XBP1 (spliced or sXBP1). sXBP1 is involved in UPR by encoding the ER chaperone genes, which increase the folding capacity of the protein in the ER lumen. Moreover, sXBP1 regulates ERAD by enhancing unfolded or misfolded protein degradation via cytosolic degradation [28, 29]. Hence, as we reported when presenting our findings, higher sXBP1 protein expression among kratom users compared to the healthy control group in our study denotes that a greater degree of UPR and ERAD occurs with chronic kratom consumption (Figs 2 and 3).

Second, in the PERK-regulated pathway, the dissociation of BiP leads to the activation of PERK via dimerization and autophosphorylation. Then, PERK regulates the phosphorylation of eIF2α to activate this regulatory node. The activated eIF2α is involved in UPR by facilitating the slowdown of the protein translation process to prevent the further accumulation of unfolded or misfolded protein in the ER lumen. Additionally, activated eIF2α regulates the translation of ATF4, which is involved in UPR by regulating the upregulation of ER chaperones to help with protein folding and autophagy in order to promote cell survival. If ER stress and UPR are protracted, ATF4 may also redirect UPR toward cell apoptosis [30, 31]. The higher ATF4 protein expression among kratom users compared to the control group in our study points to a higher degree of UPR and ER stress persistence under regular kratom consumption (Figs 2 and 3).

CHOP is a pro-apoptotic transcription factor encoded by the DNA damage-inducible transcript 3 gene. CHOP function as a dorminant-negative inhibitor by binding to other CCAAT/enhancer-binding protein (C/EBP) member to prevent their DNA binding capacity. The main role of CHOP is its involvement in ER stress induced cell apoptosis via a variety of upstream and downstream regulatory pathways. When UPR fails to counteract persistent ER stress, such that unfolded or misfolded protein continues to accumulate in the ER lumen, increase CHOP expression is induced by the integrated stress response pathway through downstream phosphorylation of the eIF2α and activation of ATF4 (function as transcription factor). In addition, activation of IRE-1α during ER stress spliced the XBP1 intron to form the activated spliced XBP1 (s-XBP1) protein. s-XBP1 protein upregulate the expression of CHOP. Elevated expression of ATF4 and CHOP protein in this study (Fig 2) indicated the possible activation of the PERK-–eIF2α–ATF4–CHOP pathway during ER stress. Elevated expression of s-XBP1 and CHOP in this study (Fig 2) indicated the possible activation of the IRE-1α—sXBP1—CHOP pathway denoting persistence of ER stress. CHOP then regulate a few downstream pathways which lead to cell apoptosis: (1) apoptosis induction via mitochondria-dependent pathway (CHOP downregulate anti-apoptotic protein B-cell lymphoma-2 (BCL2) protein and upregulate pro-apoptotic BIM, BAK and BAX proteins) and (2) apoptosis induction via death-receptor pathway (activation of PERK-–eIF2α–ATF4–CHOP pathway enable CHOP to bind to death receptors and upregulate death receptor 4 and 5). Ultimately, persistent ER stress and UPR’s failure to restore ER homeostasis result in cellular apoptosis [32–35].

JNK is kinases that phosphorylates c-Jun within its transcriptional activation domain. The activated form of JKN which is the phosphorylated JNK (p-JNK) are involved in a number of cellular activities such as apoptosis, neurodegeneration, inflammation and cytokine production, and cell differentiation and proliferation. One of its important role is its involvement in ER stress and UPR. In the presence of UPR induced by ER stress, the activated IRE—1α interact with presenilin-1 (PS1) phosphorylate JNK to its active form, p-JNK. p-JNK in turn phosphorylates and activates pro-apoptotic proteins, such as BIM and BMF. Moreover, p-JNK also phosphyrylates anti-apoptotic proteins, such as DP5-HRK, Bcl-2, and Bcl-xL, to inhibit them. Hence, elevated expression of JNK and p-JNK in this study denotes activation of the IRE-1α–JNK—p-JNK pathways of the ER stress possibly indicative of persistent ER stress and UPR’s failure to restore ER homeostasis result in cellular apoptosis [36].

As greater protein expression of CHOP and p-JNK contributed higher odds of kratom use disorder among regular kratom users after the multiple logistic regression model controlled for the protein expression levels of other ER stress sensors, kratom use characteristics, and confounding factors (age, blood pressure, and cigarette smoking); there is a possibility that persistent ER stress leading to decompensation of UPR’s capacity to restore ER homeostasis may be linked to increase risk of developing kratom use disorder. Kratom exhibited psychostimulant properties similar to methamphetamine and cocaine [37, 38]. Cocaine and methamphetamine act on the dopamine transporter at the presynaptic neuronal membrane to inhibit the reuptake of dopamine from the synapse of the mesocorticolimbic dopaminergic neuronal pathway in the brain. In turn, this inhibition increases the accumulation of dopamine. Additionally, methamphetamine increases the accumulation of dopamine in the synapse by inducing the reverse release of dopamine from the dopamine reuptake pump. This increase in extracellular dopamine is linked to inducing ER stress whereby: (1) increase dopamine release in the mesocorticolimbic dopaminergic neuronal pathway is related to enhance expression of ER stress sensors, such as BiP, PERK, and IRE1α in cocaine use and (2) accumulation of dopamine in the synapses of dopaminergic neurons is related to increase expression of BiP, CHOP, and ATF4 in methamphetamine use [10]. As for comparison, elevation of BiP in cocaine and methamphetamine use indicates activation of ER stress. While elevation of PERK, IRE1α, ATF4, and CHOP denotes persistent of ER stress and activation of the PERK–eIF2α–ATF4–CHOP and IRE1α–JNK—p-JNK pathways of ER stress which may decompensate UPR’s capacity to restore ER homeostasis, possibly progressing towards cell apoptosis. Hence, the findings of ER stress in cocaine and methamphetamine use are parallel to the findings in this study. Moreover, cocaine may produce recurrent defective cystine or glutamate exchange and glutamate transport, which impair glutamate extracellular homeostasis, leading to cocaine’s addictive qualities and relating to cocaine-induced ER stress. Hence in short and medium term of psychostimulant consumption, it induced behavioral sensitization and in long term, it may cause neurodegeneration in a manner similar to neurodegenerative disease like Alzheimer’s disease [10].

Similarly, the accumulation of dopamine and glutamate in the synapse of neurons in the mesocorticolimbic dopaminergic neuronal pathway, which induced persistent ER stress and UPR (elevated expression of CHOP and p-JNK associated with kratom use disorder) may also apply to kratom alkaloids, such as mitragynine and 7-HMG, which act as μ-opioid receptor agonists and induce extracellular dopamine and glutamate accumulation in the brain [39]. Hence, repeated kratom consumption in short and medium term may increase risk of behavioral sensitization and in long term induced neurodegeneration.

Our findings should be interpreted while considering a few of our study’s limitations. First, this study recruited participants only from Penang State in the northern region of Peninsular Malaysia. This restriction limits our findings’ generalizability since our study may not represent the entire kratom user population in Malaysia. Second, although our findings may point toward the activation of the PERK–eIF2α–ATF4–CHOP and IRE1α–JNK—p-JNK pathways of ER stress, this study did not assess some of the ER stress sensors involved in UPR cascades, such as PERK, IREα, ATF6, and eIF2α. Hence, determining how these ER stress sensors alter in relation to regular kratom exposure and kratom dependence severity requires further investigation. Finally, the study findings at its best indicated that persistent ER stress and UPR may be associated with kratom use disorder, but it did not pinpoint whether persistent ER stress and UPR is a causative factor to occurrence of kratom use disorder. To date, it is unclear whether persistent ER stress and UPR is a causative mechanism underlying occurrence of substance use disorder or rather as a cellular outcome of substance use disorder. Hence, we recommend future cohort or prospective studies to investigate on this research gap to further shed light on the understanding of the relationship between ER stress and kratom or other substance use disorder.

This study’s strengths include a comprehensive investigation of how human participants’ regular kratom use affected ER stress and how alteration in the ER stress chaperones was associated with kratom use disorder. This study provided important data on the extent to which ER stress occurred in relation to daily kratom consumption and whether persistent ER stress might lead to UPR decompensation in order to regain ER homeostasis, which may contribute to kratom use disorder. Moreover, intensive clinical screening was conducted to ensure that all recruited participants were not influenced by medical or psychiatric illnesses’, substances’, or medications’ effects on ER stress. This process included thoroughly screening for medical and psychiatric illnesses, as well as past and current histories of drug, medication, and alcohol use.

## Conclusion

This study provides useful data indicating that alterations in the protein expression of ER stress sensors, such as BiP, sXBP1, ATF4, CHOP, and JNK, may serve as biomarkers with which to evaluate kratom use disorder, providing valuable information for clinicians treating chronic kratom use. These potential biomarkers may also provide clinicians with useful data on the efficacy of treatment for kratom use disorder, possibly showing that effective treatment may reverse the alteration of ER stress sensors. For example, how well people with kratom use disorder respond to treatment with Suboxone [buprenorphine and naloxone] [40] by comparing ER stress sensors before and after treatment would be interesting to investigate. We also recommend that a future study compare whether any differences in ER stress alteration arise between individuals who use only kratom and those who use both kratom and other substances to determine whether kratom’s co-administration with other substances could exacerbate ER stress. In addition, since a large population of opioid users are also using kratom as a self-prescribed replacment agent to self-treat opioid use disorder, we plan to conduct a follow-up study on the cost-effectiveness of kratom use in the opioid using population as kratom may also induce ER stress and kratom use disorder as well.

## Supporting information

S1 Appendix Kratom use characteristics questionnaire (for regular kratom users only). (DOCX)

S2 Appendix Sociodemographic and clinical characteristics questionnaire (for all participants). (DOCX)

S3 Appendix Publication and licensing rights for Fig 3. (DOCX)

S1 Fig The raw images of the Western blot analysis of the targeted ER stress sensors in this study. (PDF)

S1 Checklist STROBE statement—Checklist of items that should be included in reports of observational studies. (DOCX)

S1 Table The blood investigation of the participants. (DOCX)

S2 Table Socio-demographic and clinical characteristics of participants. (DOCX)

S3 Table Characteristics of kratom use among the regular kratom users. (DOCX)
