# Supplementary material for: Kratom use disorder and unfolded protein response: Evaluating their relationship in a case control study
Source: PLoS One. 2023 Jun 23;18(6):e0287466. doi: 10.1371/journal.pone.0287466 (PMC10289391; doi:10.1371/journal.pone.0287466)
Supplement: S1 Appendix — (DOCX) [file pone.0287466.s001.docx]

**S1 appendix**

**Kratom use characteristics questionnaire (for regular kratom users only):**

Instruction: Please answer all the questions below by ticking the appropriate response option in the box provided.

1. How long have you been consuming kratom?

eeeeeeee

For the past 1 to 6 years For more than 6 years

1. On average, how frequent do you drink kratom juice on a daily basis?

1 to 3 times per day More than 3 times per day

1. On average, how many glasses of kratom juice do you drink on a daily basis?

1 to 3 glasses per day (1 glass equivalent to approximately 300 ml)

More than 3 glasses per day (1 glass equivalent to approximately 300 ml)
