# Supplementary material for: Kratom use disorder and unfolded protein response: Evaluating their relationship in a case control study
Source: PLoS One. 2023 Jun 23;18(6):e0287466. doi: 10.1371/journal.pone.0287466 (PMC10289391; doi:10.1371/journal.pone.0287466)
Supplement: S2 Appendix — (DOCX) [file pone.0287466.s002.docx]

**S2 appendix**

**Sociodemographic and clinical characteristics questionnaire (for all participants):**

Instruction: Please answer all the questions below by ticking on the appropriate response option provided in the box.

1. Your age?

< 40 years old ≥ 40 years old

1. Your ethnicity?

Malay Non-Malay

1. Your employment status?

Employed Non-employed/student

1. Your education level?

Up to secondary education Tertiary education

1. Your monthly household income?

≤ RM 1000 > RM 1000

1. Your marital status?

Married Single/divorce/widower

1. Your body mass index (BMI)?

Weight (in kg): Height (in metre):

BMI = weight (in kg) =

(height)^2^ (in m^2^)

1. Do you smoke?

I am a non-smoker I smoke 10 sticks/day

I smoke more than 10 sticks/day

1. Your blood pressure?

Normal (< 130/80 mmHg) High (≥ 130/80 mmHg)

1. Your pulse rate?

Normal (< 100 beats/minute) High (≥ 100 beats/minute)
