# Supplementary figures and images for: Kratom use disorder and unfolded protein response: Evaluating their relationship in a case control study
Source: PLoS One. 2023 Jun 23;18(6):e0287466. doi: 10.1371/journal.pone.0287466 (PMC10289391; doi:10.1371/journal.pone.0287466)

**S3 appendix (Publication and Licensing Rights for Fig 3):**


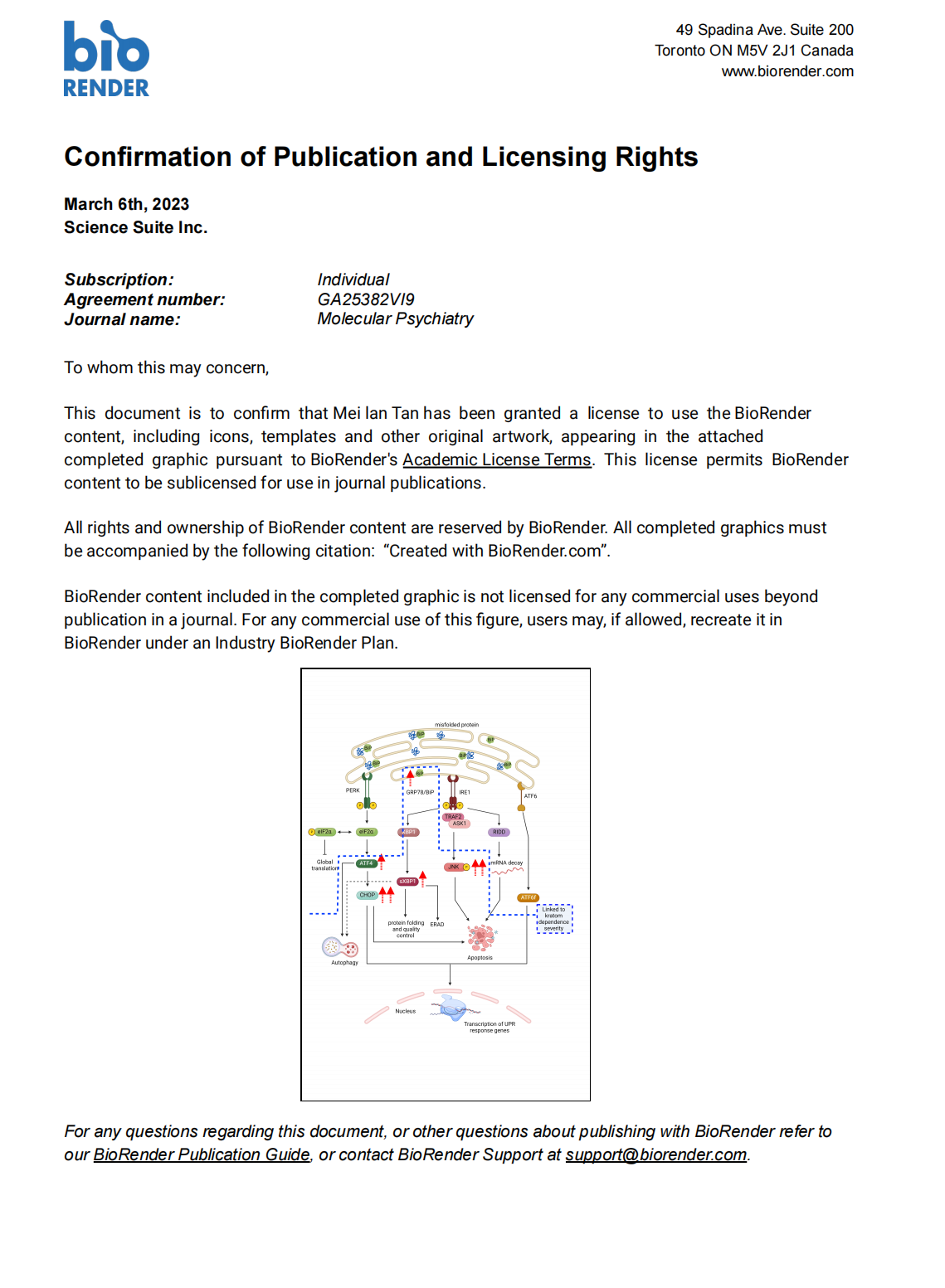

Supplement: S3 Appendix — (DOCX) [file pone.0287466.s003.docx]
