# Supplementary material for: Kratom use disorder and unfolded protein response: Evaluating their relationship in a case control study
Source: PLoS One. 2023 Jun 23;18(6):e0287466. doi: 10.1371/journal.pone.0287466 (PMC10289391; doi:10.1371/journal.pone.0287466)
Supplement: S1 Fig — (PDF) [file pone.0287466.s004.pdf]

The original blot of BIP in Fig. 2

All images were captured by VersaDoc™ MP Imaging Systems (Bio-Rad Laboratories, USA).

From 1 to 5, 11 to 15, 21 to 25, 31 to 35, 41 to 45, 51 to 55, 61 to 65, 71 to 75, 81 to 85, and 91 to 95: control subjects.

From 6 to 10, 16 to 20, 26 to 30, 36 to 40, 46 to 50, 56 to 60, 66 to 70, 76 to 80, 86 to 90, 96 to 100, and 101 to 110: regular kratom users.

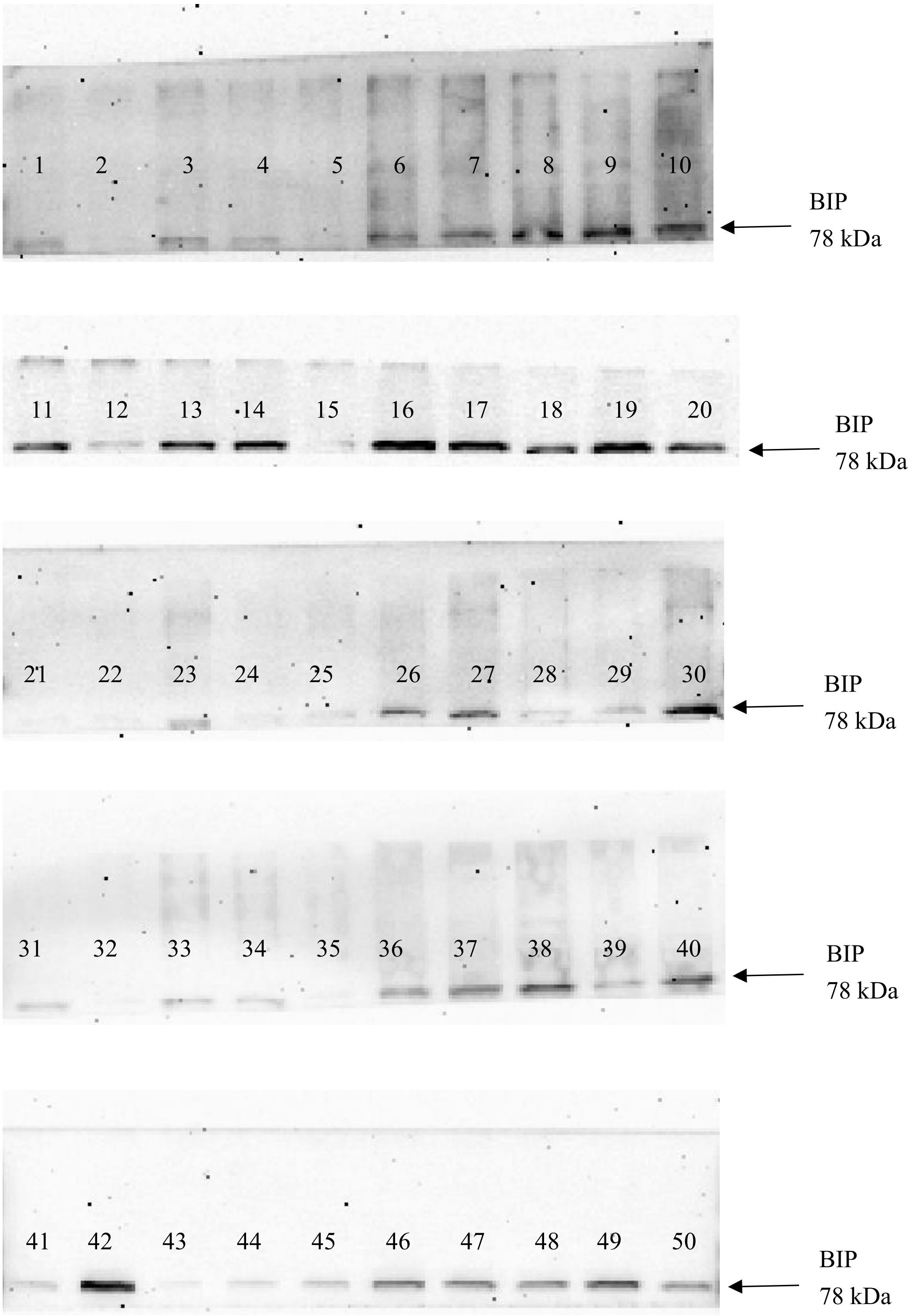

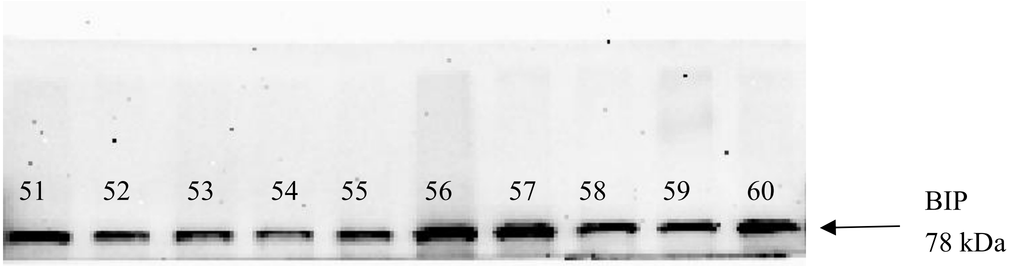
51 52 53 54 55 56 57 58 59 60
BIP
78 kDa

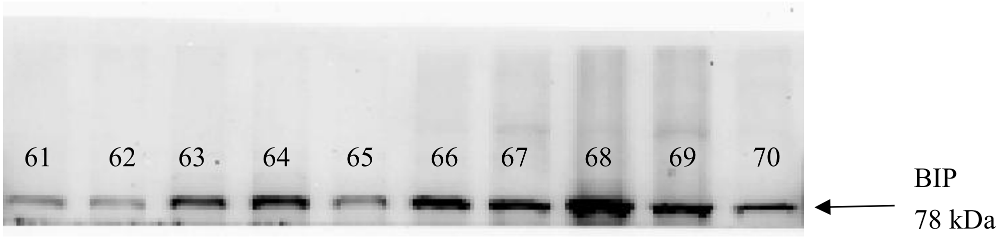
61 62 63 64 65 66 67 68 69 70
BIP
78 kDa

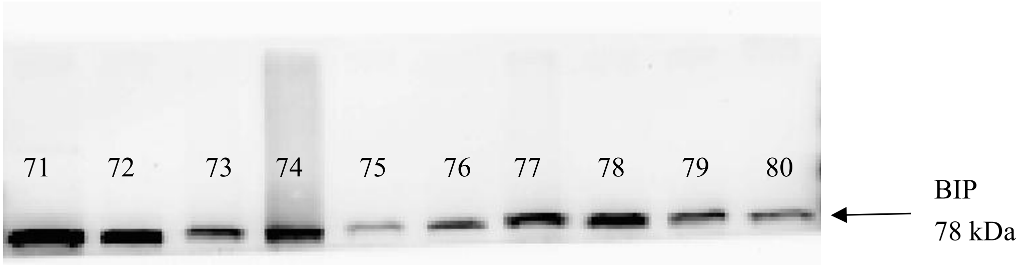
71 72 73 74 75 76 77 78 79 80
BIP
78 kDa

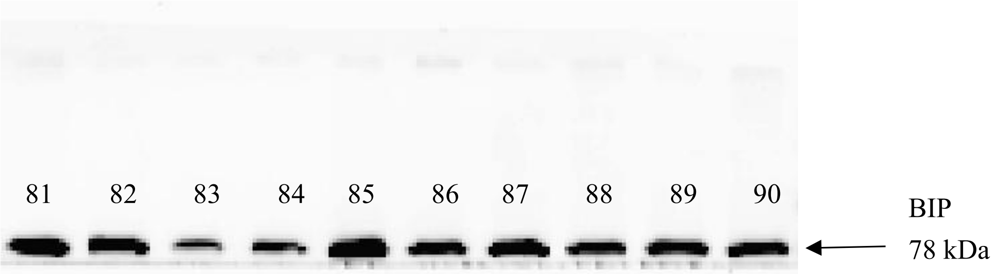
81 82 83 84 85 86 87 88 89 90
BIP
78 kDa

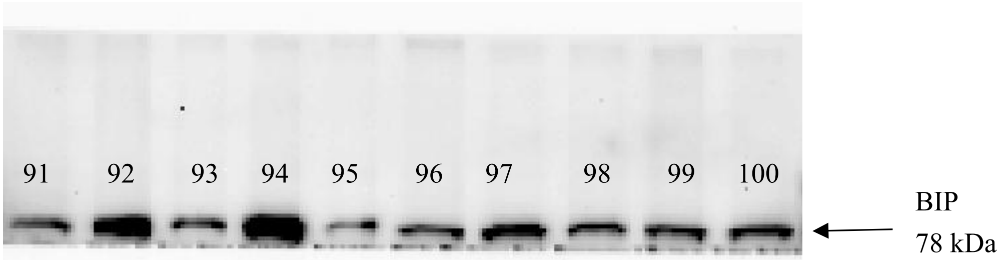
91 92 93 94 95 96 97 98 99 100
BIP
78 kDa

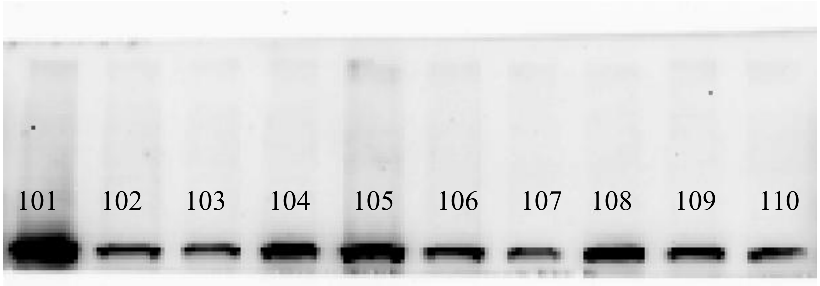

BIP
78 kDa

The original blot of XBP1s in Fig. 2

All images were captured by VersaDoc™ MP Imaging Systems (Bio-Rad Laboratories, USA).

From 1 to 5, 11 to 15, 21 to 25, 31 to 35, 41 to 45, 51 to 55, 61 to 65, 71 to 75, 81 to 85, and 91 to 95: control subjects.

From 6 to 10, 16 to 20, 26 to 30, 36 to 40, 46 to 50, 56 to 60, 66 to 70, 76 to 80, 86 to 90, 96 to 100, and 101 to 110: regular kratom users.

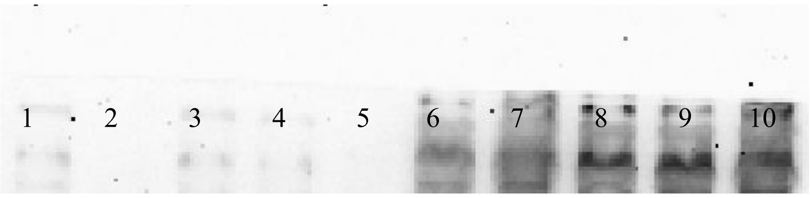

XBP1s
60 kDa

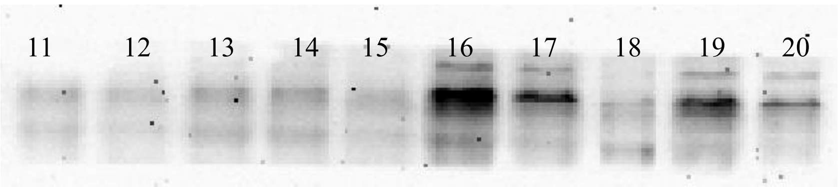

XBP1s
60 kDa

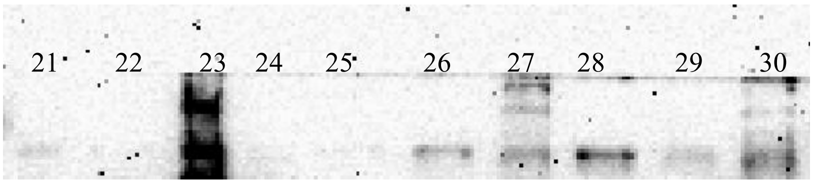

XBP1s
60 kDa

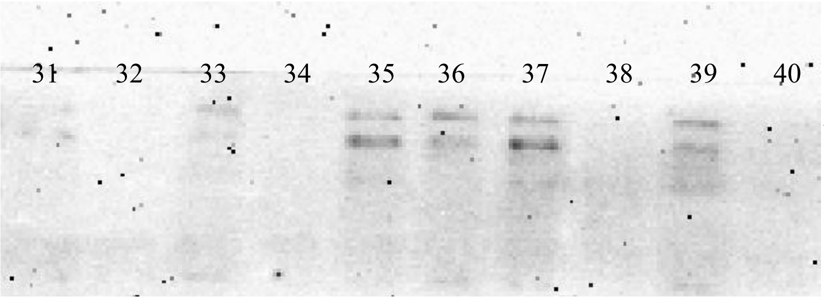

XBP1s
60 kDa

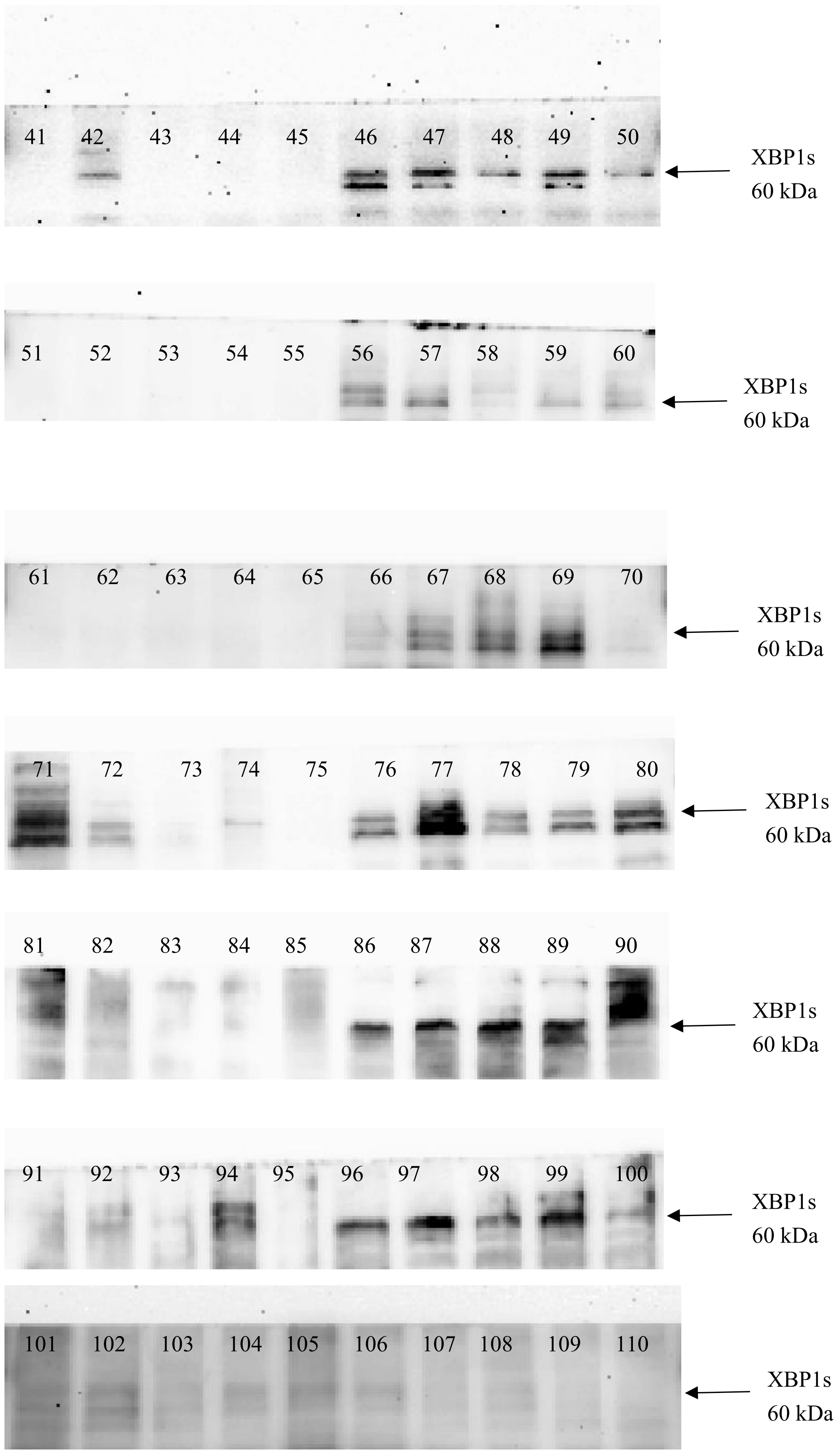
41 42 43 44 45 46 47 48 49 50
XBP1s
60 kDa
51 52 53 54 55 56 57 58 59 60
XBP1s
60 kDa
61 62 63 64 65 66 67 68 69 70
XBP1s
60 kDa
71 72 73 74 75 76 77 78 79 80
XBP1s
60 kDa
81 82 83 84 85 86 87 88 89 90
XBP1s
60 kDa
91 92 93 94 95 96 97 98 99 100
XBP1s
60 kDa
101 102 103 104 105 106 107 108 109 110
XBP1s
60 kDa

The original blot of ATF4 in Fig. 2

All images were captured by VersaDoc™ MP Imaging Systems (Bio-Rad Laboratories, USA).

From 1 to 5, 11 to 15, 21 to 25, 31 to 35, 41 to 45, 51 to 55, 61 to 65, 71 to 75, 81 to 85, and 91 to 95: control subjects.

From 6 to 10, 16 to 20, 26 to 30, 36 to 40, 46 to 50, 56 to 60, 66 to 70, 76 to 80, 86 to 90, 96 to 100, and 101 to 110: regular kratom users.

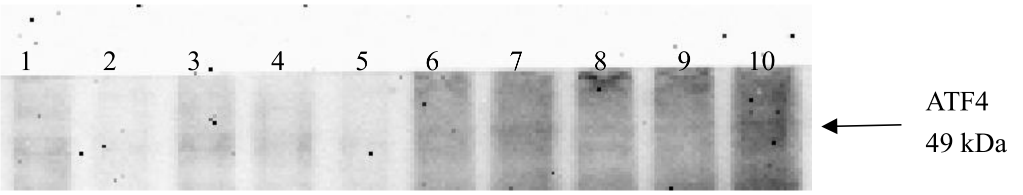

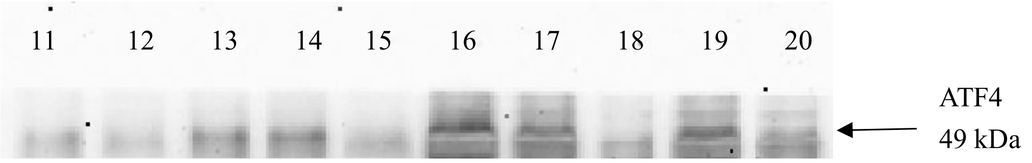

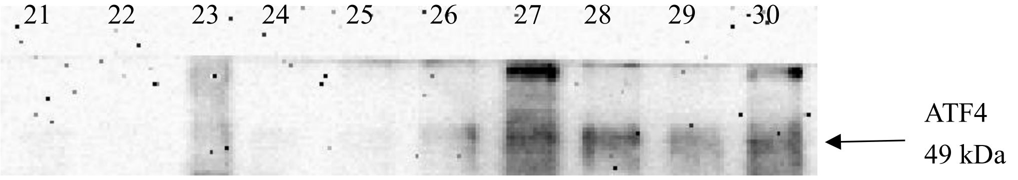

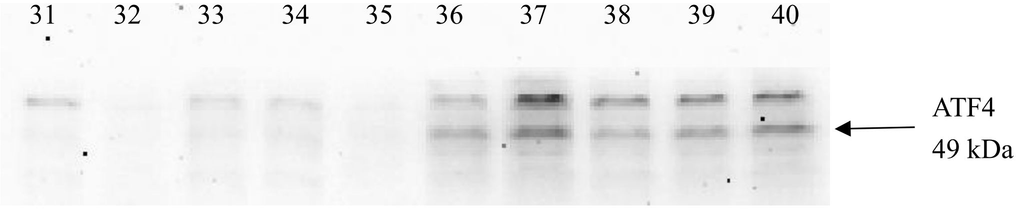

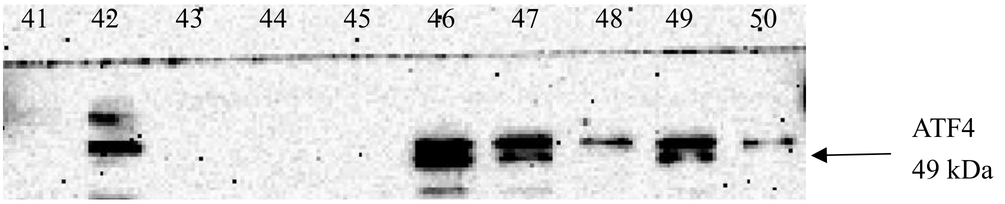

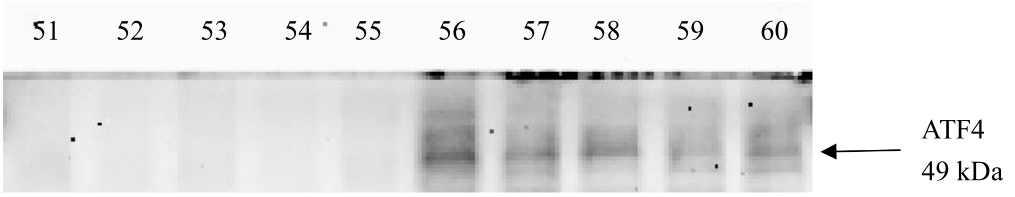

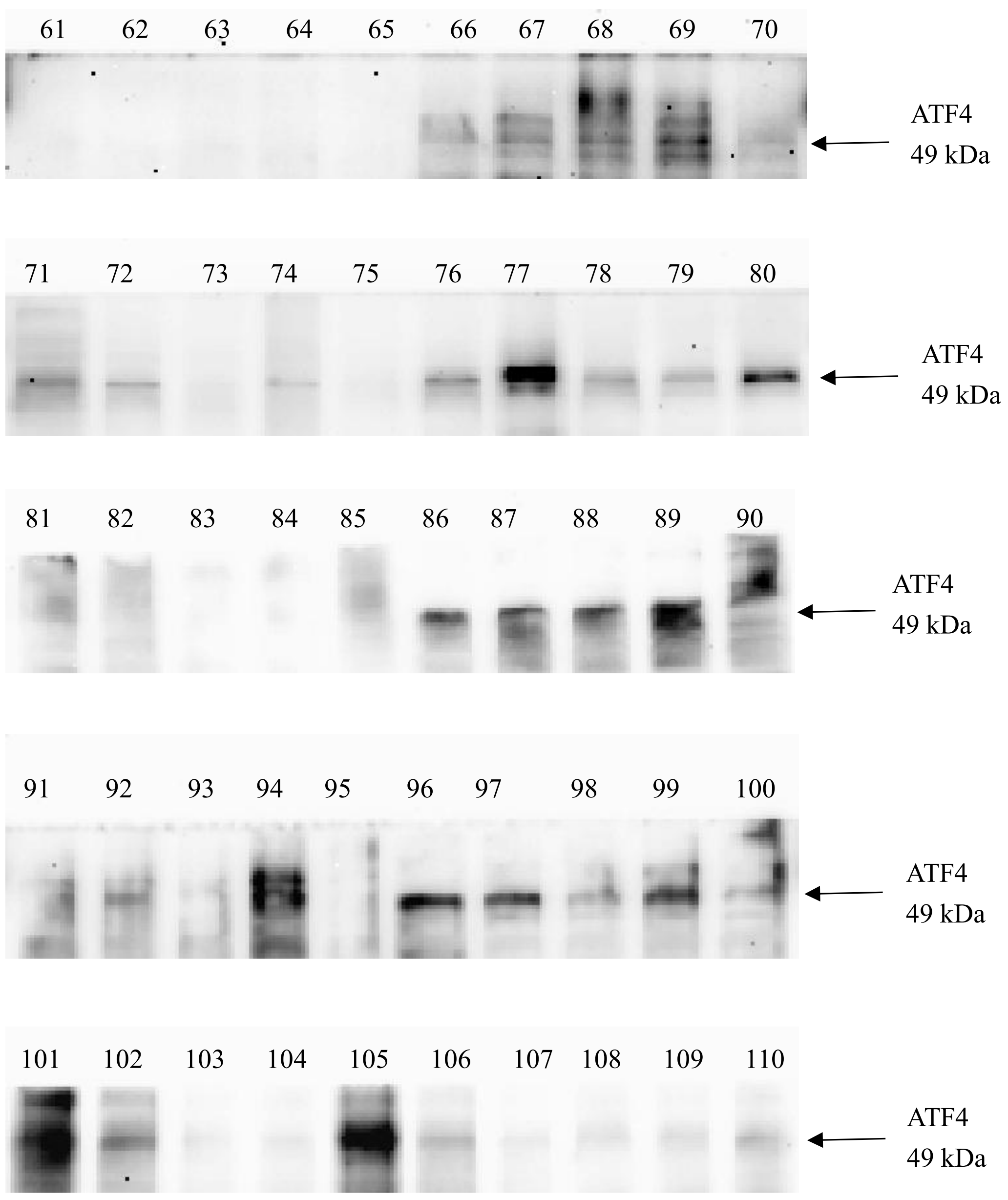

The original blot of CHOP in Fig. 2

All images were captured by VersaDoc™ MP Imaging Systems (Bio-Rad Laboratories, USA).

From 1 to 5, 11 to 15, 21 to 25, 31 to 35, 41 to 45, 51 to 55, 61 to 65, 71 to 75, 81 to 85, and 91 to 95: control subjects.

From 6 to 10, 16 to 20, 26 to 30, 36 to 40, 46 to 50, 56 to 60, 66 to 70, 76 to 80, 86 to 90, 96 to 100, and 101 to 110: regular kratom users.

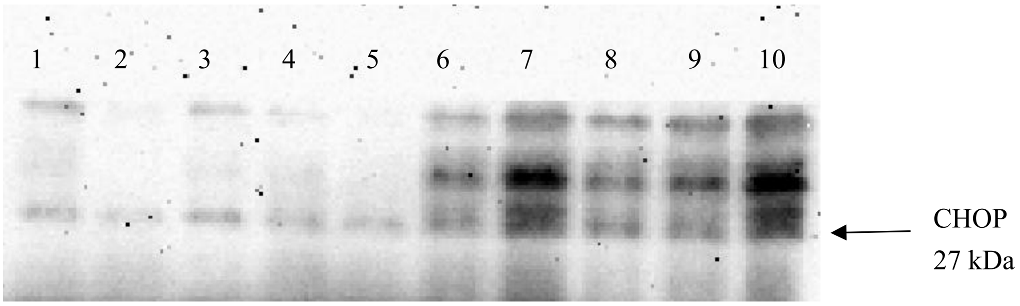
1 2 3 4 5 6 7 8 9 10
CHOP
27 kDa

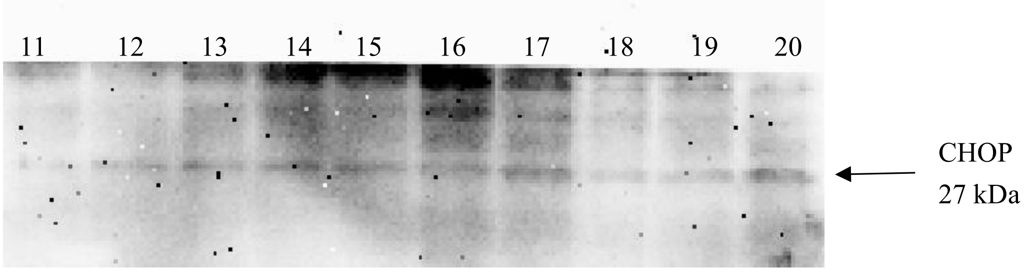
11 12 13 14 15 16 17 18 19 20
CHOP
27 kDa

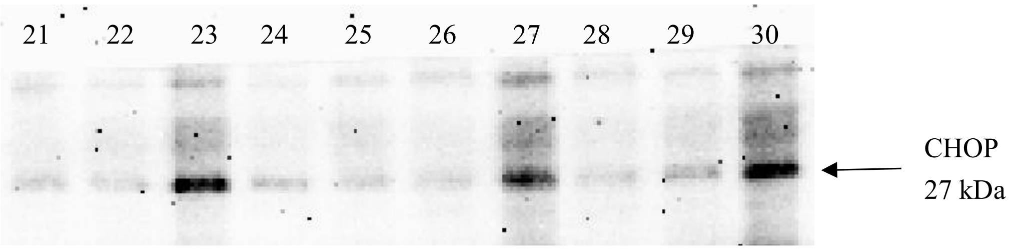
21 22 23 24 25 26 27 28 29 30
CHOP
27 kDa

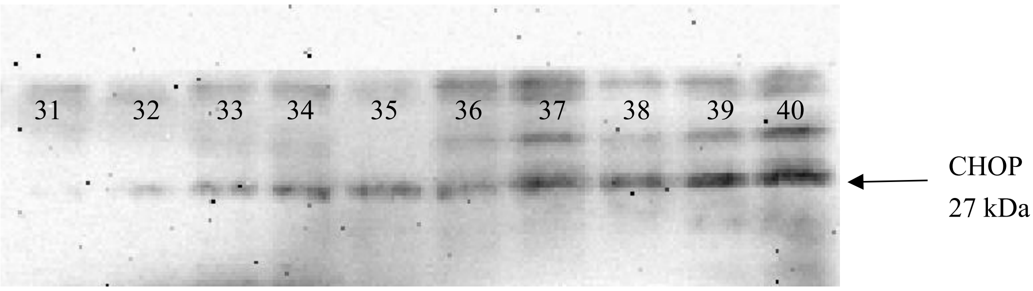
31 32 33 34 35 36 37 38 39 40
CHOP
27 kDa

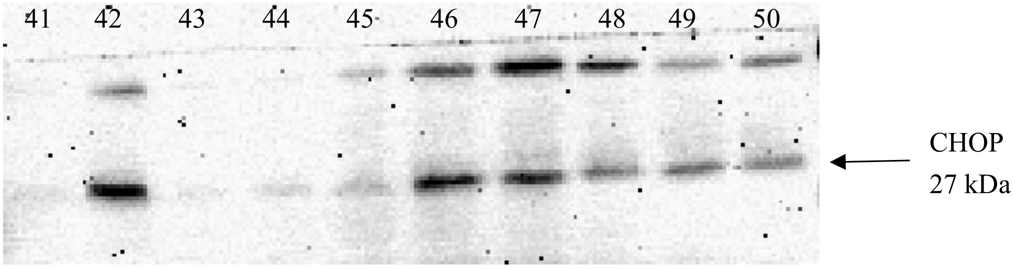
41 42 43 44 45 46 47 48 49 50
CHOP
27 kDa

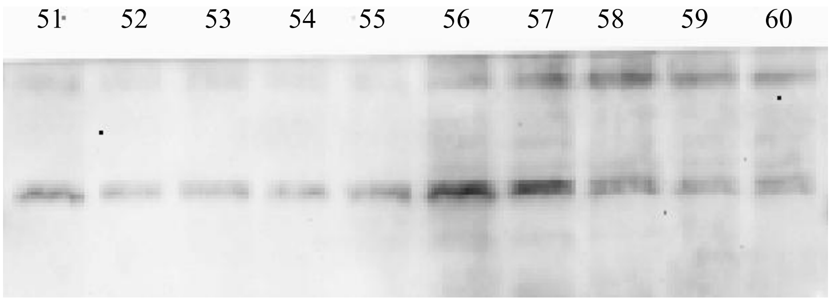
51 52 53 54 55 56 57 58 59 60
CHOP
27 kDa

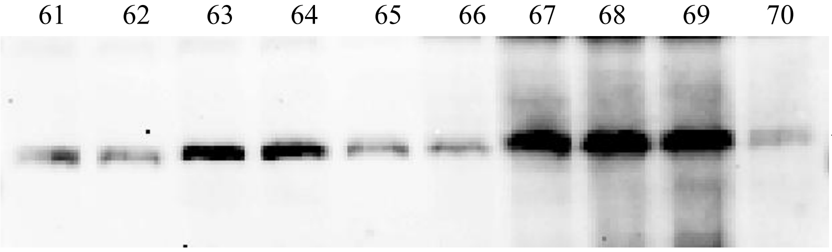
61 62 63 64 65 66 67 68 69 70
CHOP
27 kDa

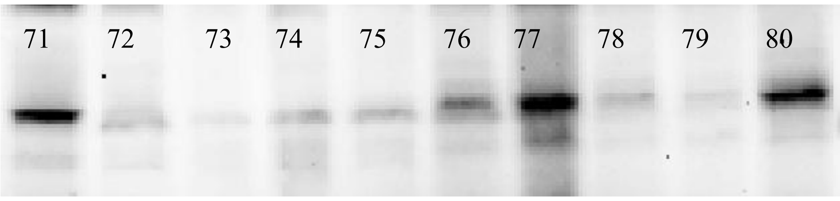
71 72 73 74 75 76 77 78 79 80
CHOP
27 kDa

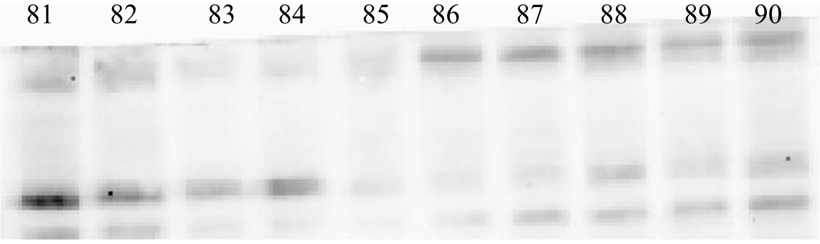
81 82 83 84 85 86 87 88 89 90
CHOP
27 kDa

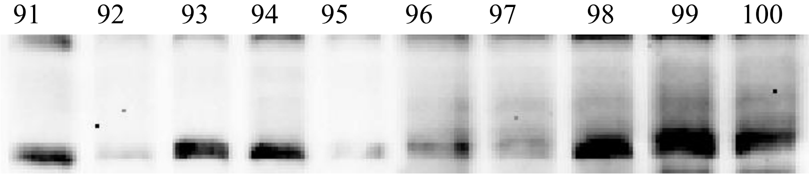
91 92 93 94 95 96 97 98 99 100
CHOP
27 kDa

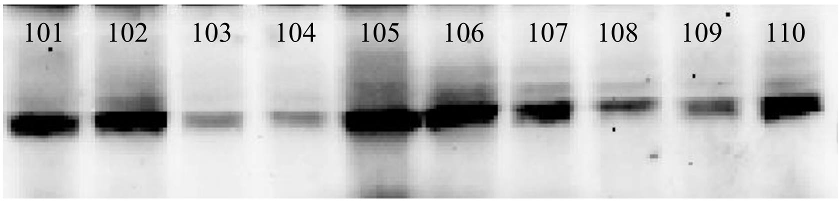
101 102 103 104 105 106 107 108 109 110
CHOP
27 kDa

The original blot of P-JNK in Fig. 2

All images were captured by VersaDoc™ MP Imaging Systems (Bio-Rad Laboratories, USA).

From 1 to 5, 11 to 15, 21 to 25, 31 to 35, 41 to 45, 51 to 55, 61 to 65, 71 to 75, 81 to 85, and 91 to 95: control subjects.

From 6 to 10, 16 to 20, 26 to 30, 36 to 40, 46 to 50, 56 to 60, 66 to 70, 76 to 80, 86 to 90, 96 to 100, and 101 to 110: regular kratom users.

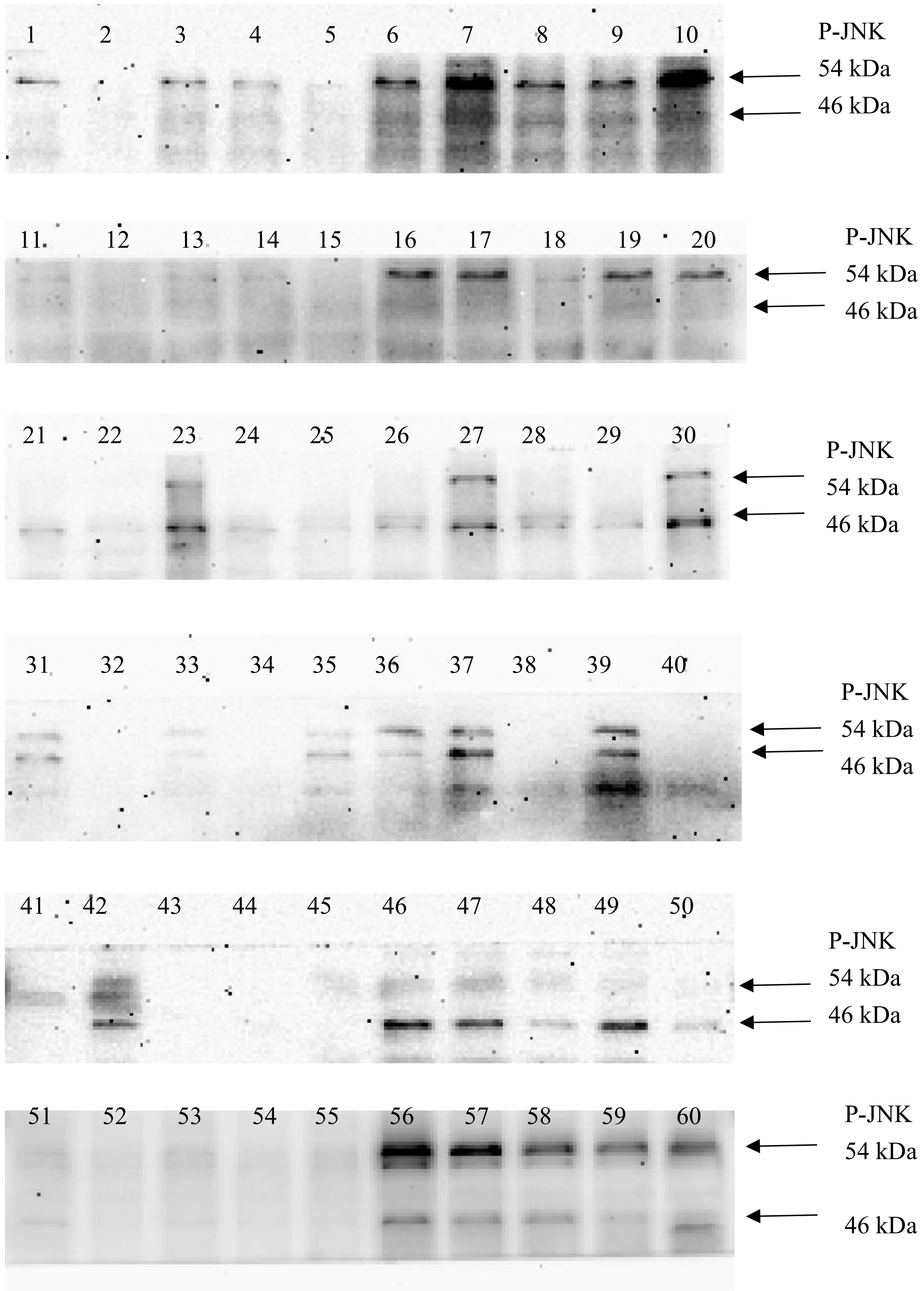

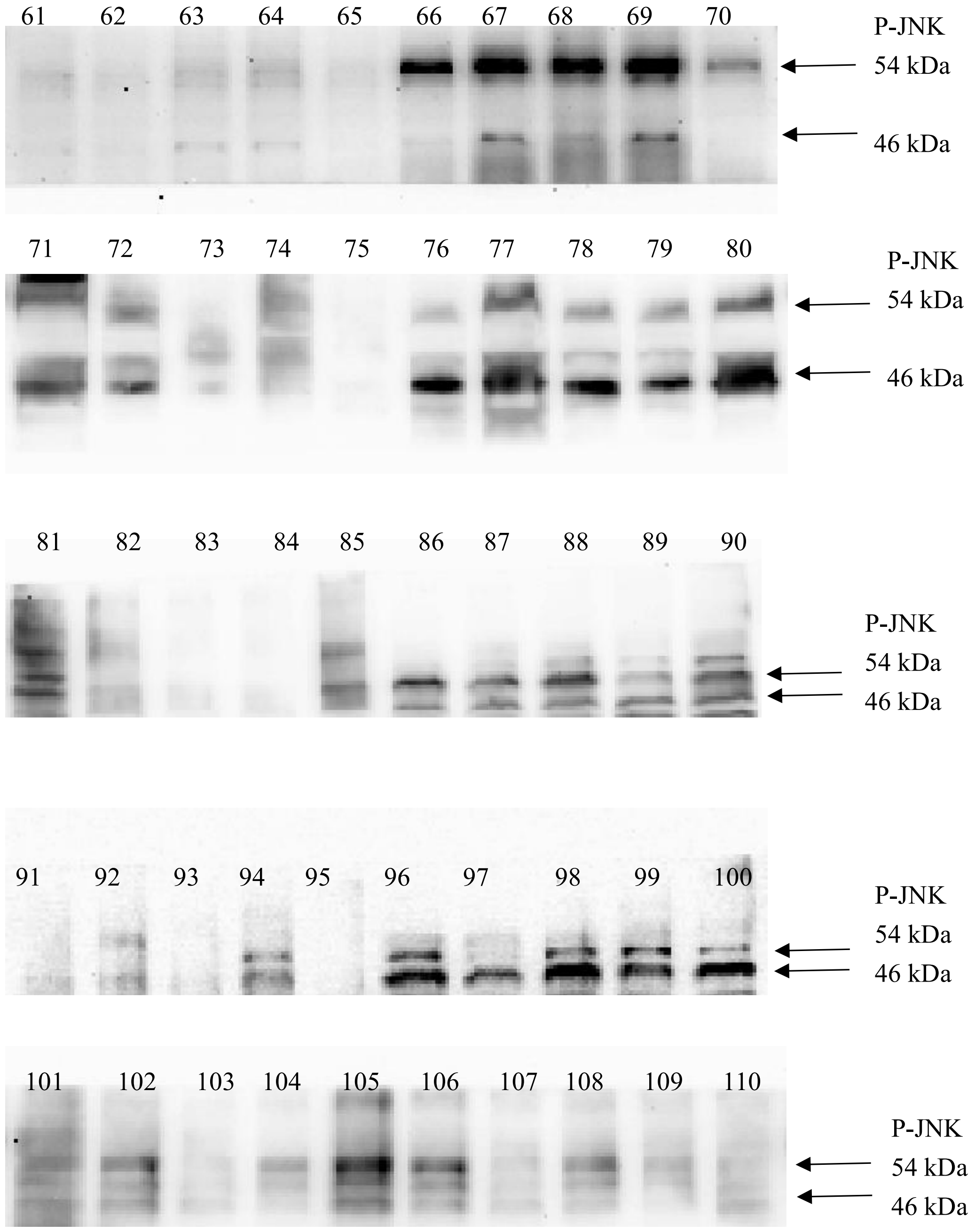
61 62 63 64 65 66 67 68 69 70
P-JNK
54 kDa
46 kDa
71 72 73 74 75 76 77 78 79 80
P-JNK
54 kDa
46 kDa
81 82 83 84 85 86 87 88 89 90
P-JNK
54 kDa
46 kDa
91 92 93 94 95 96 97 98 99 100
P-JNK
54 kDa
46 kDa
101 102 103 104 105 106 107 108 109 110
P-JNK
54 kDa
46 kDa

The original blot of JNK in Fig. 2

All images were captured by VersaDoc™ MP Imaging Systems (Bio-Rad Laboratories, USA).

From 1 to 5, 11 to 15, 21 to 25, 31 to 35, 41 to 45, 51 to 55, 61 to 65, 71 to 75, 81 to 85, and 91 to 95: control subjects.

From 6 to 10, 16 to 20, 26 to 30, 36 to 40, 46 to 50, 56 to 60, 66 to 70, 76 to 80, 86 to 90, 96 to 100, and 101 to 110: regular kratom users.

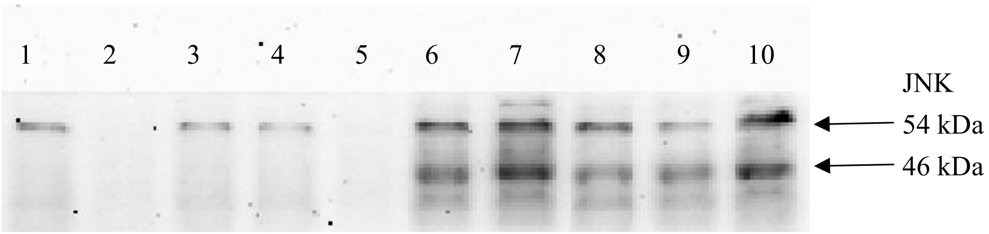

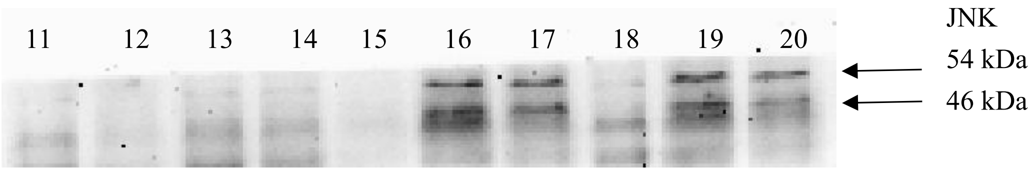

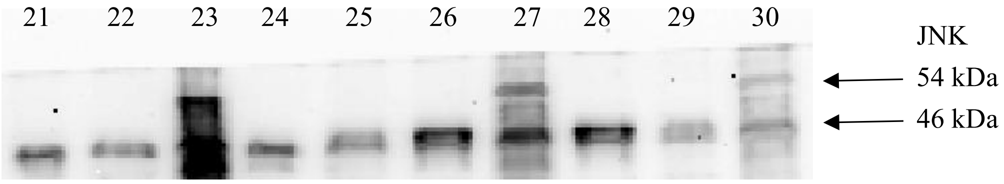

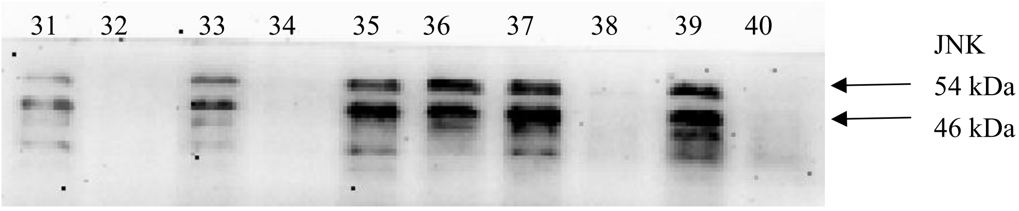

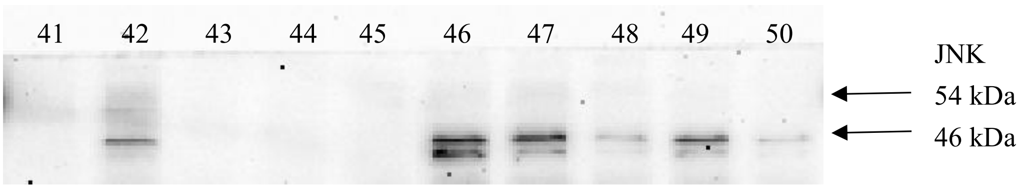

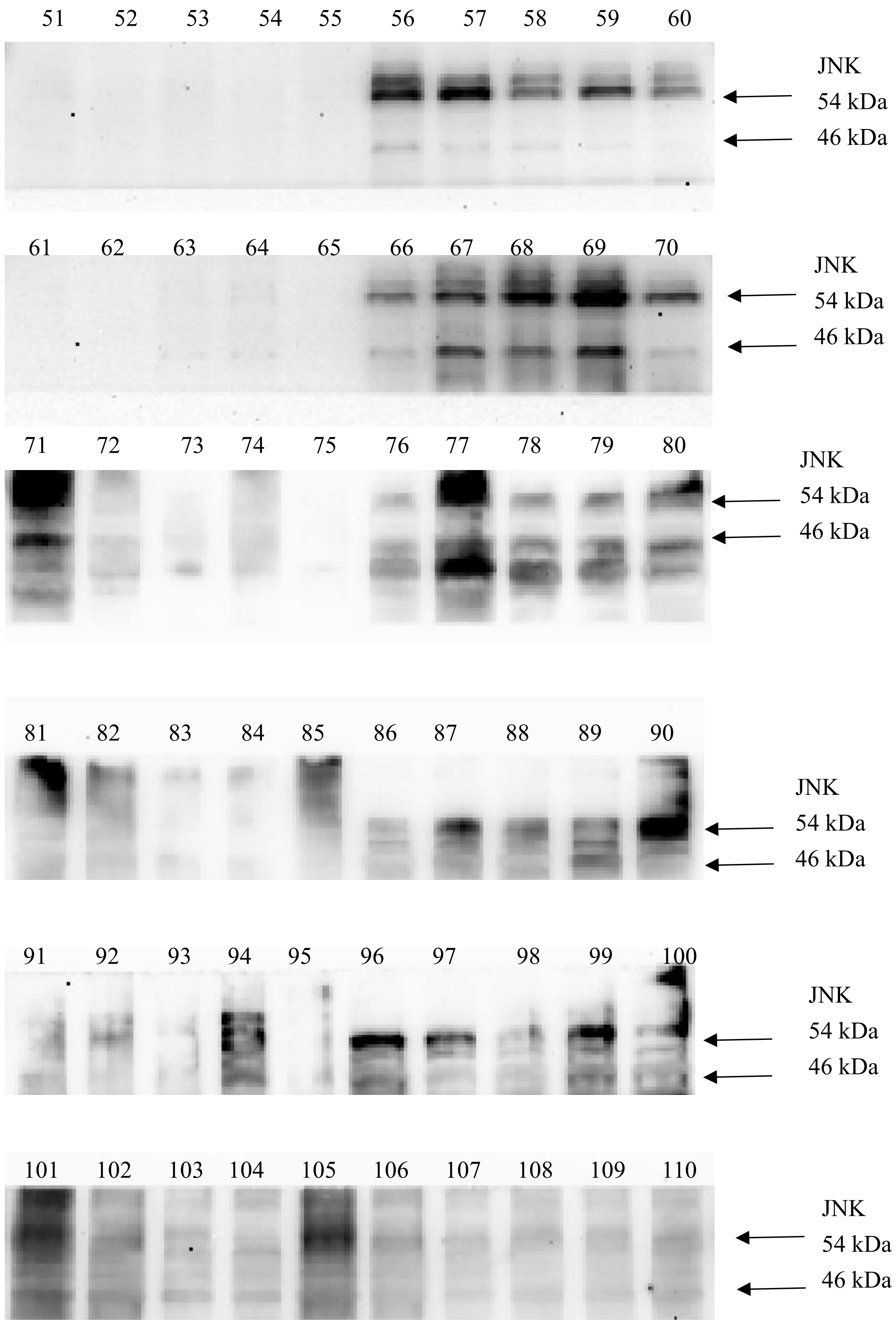
51 52 53 54 55 56 57 58 59 60
JNK
54 kDa
46 kDa
61 62 63 64 65 66 67 68 69 70
JNK
54 kDa
46 kDa
71 72 73 74 75 76 77 78 79 80
JNK
54 kDa
46 kDa
81 82 83 84 85 86 87 88 89 90
JNK
54 kDa
46 kDa
91 92 93 94 95 96 97 98 99 100
JNK
54 kDa
46 kDa
101 102 103 104 105 106 107 108 109 110
JNK
54 kDa
46 kDa

The original blot of β-actin in Fig. 2
All images were captured by VersaDoc™ MP Imaging Systems (Bio-Rad Laboratories, USA).
From 1 to 5, 11 to 15, 21 to 25, 31 to 35, 41 to 45, 51 to 55, 61 to 65, 71 to 75, 81 to 85, and 91 to 95: control subjects.
From 6 to 10, 16 to 20, 26 to 30, 36 to 40, 46 to 50, 56 to 60, 66 to 70, 76 to 80, 86 to 90, 96 to 100, and 101 to 110: regular kratom users.

1 2 3 4 5 6 7 8 9 10

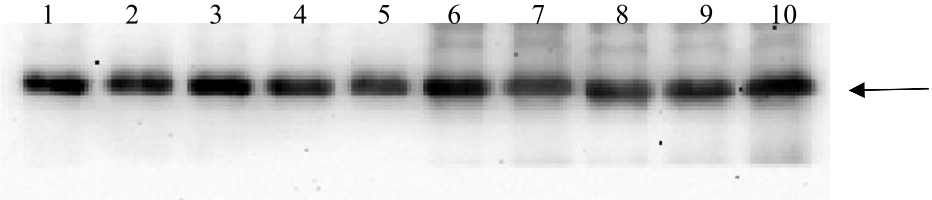

β-actin
42 kDa

11 12 13 14 15 16 17 18 19 20

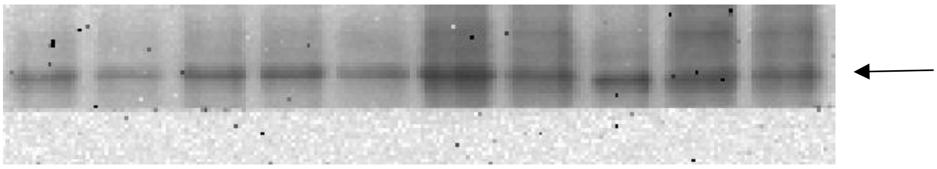

β-actin
42 kDa

21 22 23 24 25 26 27 28 29 30

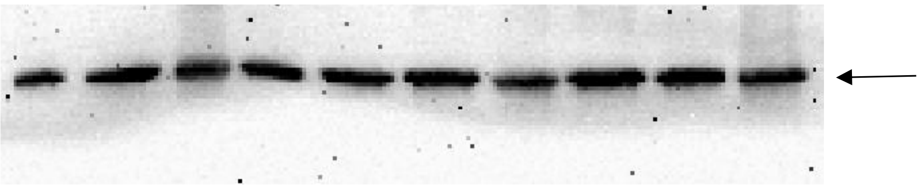

β-actin
42 kDa

31 32 33 34 35 36 37 38 39 40

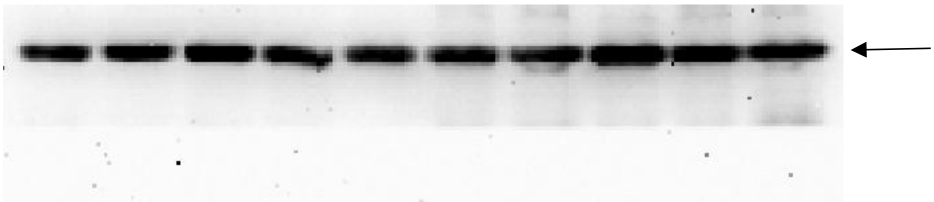

β-actin
42 kDa

41 42 43 44 45 46 47 48 49 50

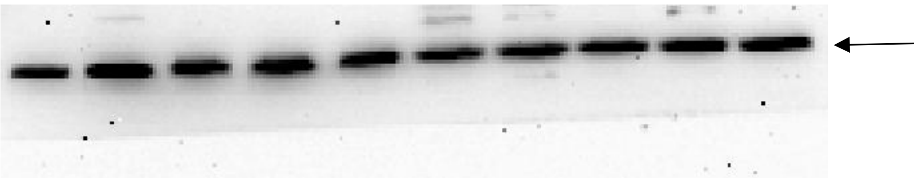

β-actin
42 kDa

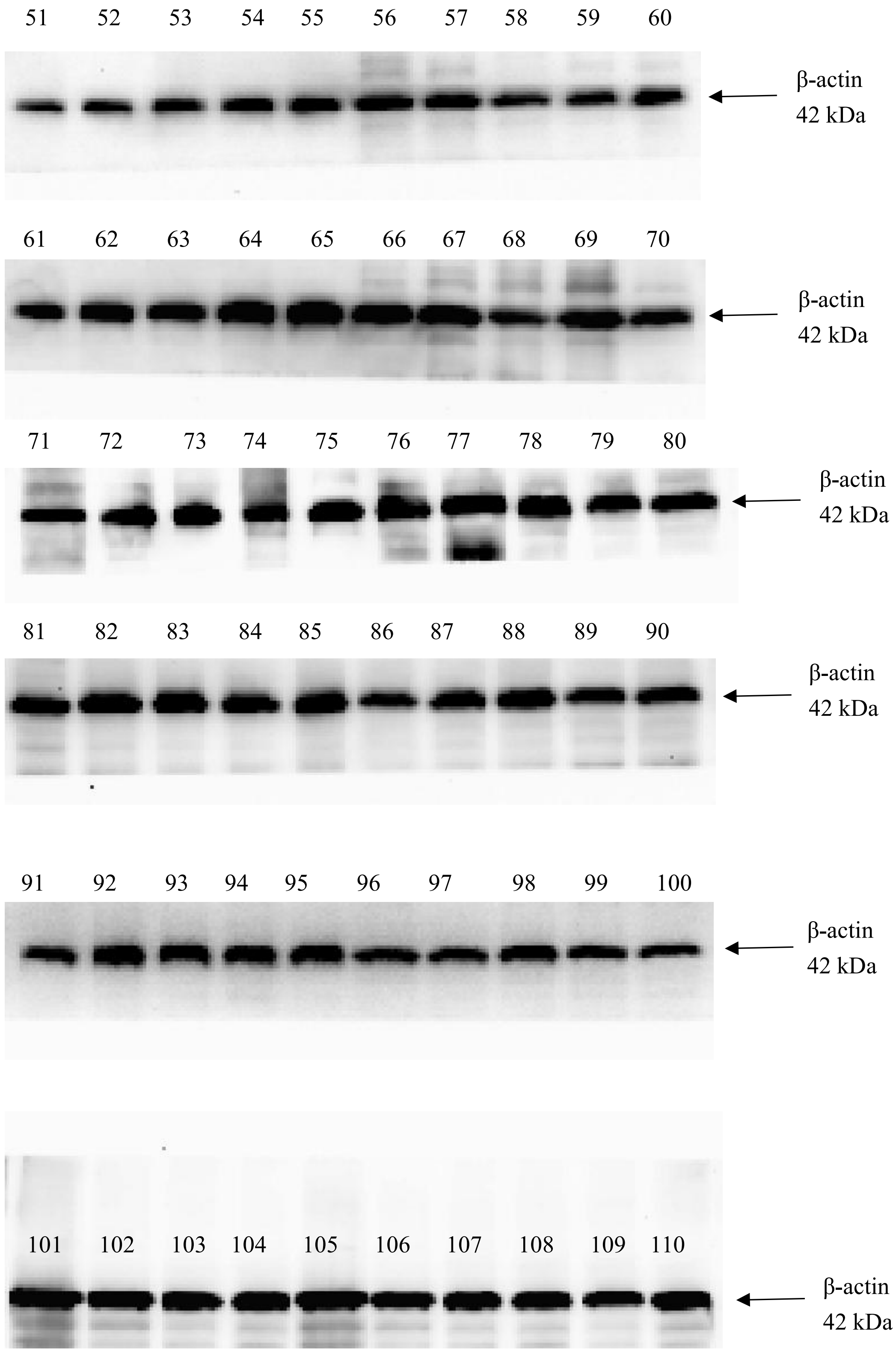

51 52 53 54 55 56 57 58 59 60
β-actin
42 kDa
61 62 63 64 65 66 67 68 69 70
β-actin
42 kDa
71 72 73 74 75 76 77 78 79 80
β-actin
42 kDa
81 82 83 84 85 86 87 88 89 90
β-actin
42 kDa
91 92 93 94 95 96 97 98 99 100
β-actin
42 kDa
101 102 103 104 105 106 107 108 109 110
β-actin
42 kDa
