# Supplementary material for: Kratom use disorder and unfolded protein response: Evaluating their relationship in a case control study
Source: PLoS One. 2023 Jun 23;18(6):e0287466. doi: 10.1371/journal.pone.0287466 (PMC10289391; doi:10.1371/journal.pone.0287466)
Supplement: S1 Table — (DOCX) [file pone.0287466.s006.docx]

**Table S1. The blood investigation of the participants**

| Variables | Kratom users  Median (IQR) | Control subjects  Median (IQR) | Normal range | Mann-Whitney U test (p-value) |
| --- | --- | --- | --- | --- |
| **Full blood count:**  Total white blood cell (x 10^3^ cells/L)  Hemoglobin (g/dL)  Platelet (x 10^3^ cells/L)  Hematocrit (%)  MCV (fL)  MCH (pg)  MCHC (g/L)  **Liver function test:**  Total protein (g/L) Albumin g/L)  Globulin (g/L)  AG ratio  Total bilirubin (µmol/L)  AST (U/L)  ALT (U/L)  ALP (U/L)  **Renal profile and electrolytes:**  Sodium (mmol/L)  Potassium (mmol/L)  Chloride (mmol/L)  Urea (mmol/L)  Creatinine (µmol/L) Calcium (mmol/L) Phosphate (mmol/L) Uric acid (µmol/L)  Thyroid function test:  T4 (nmol/L)  TSH (mIU/L)  **Fasting lipid profile and fasting blood sugar:**  Total cholesterol (mmol/L)  LDL (mmol/L)  HDL (mmol/L)  TG (mmol/L)  FBG (mmol/L) | 10.75 (4.7)  13.90 (1.3)  289.0 (100.3)  40.1 (3.2)  83.95 (6.8)  27.85 (2.7)  32.65 (0.8)  75.25 (3.38)  41.63 (1.06)  33.63 (3.20)  1.25 (0.12)  9.75 (1.67)  19.25 (1.19)  20.63 (2.17)  90.88 (8.03)  137.5 (6.0)  4.2 (0.6)  103 (4)  3.3 (3.0)  91.0 (18.5)  2.27 (0.06)  1.31 (0.23)  419.5 (160.0)  113.0 (24.3)  1.7 (1.3)  4.80 (1.33)  2.97 (1.39)  1.23 (0.36)  1.57 (1.97)  4.10 (0.85) | 8.17 (1.4)  14.80 (1.8)  253.5 (55.5)  46.3 (5.5)  83.00 (9.7)  26.80 (3.5)  32.9 (1.2)  76.63 (3.28)  43.13 (2.48)  31.75 (3.01)  1.44 (0.17)  12.38 (1.46)  26.38 (3.45)  29.50 (2.18)  98.88 (5.83)  138.0 (2.3)  4.2 (0.8)  102 (3)  5.1 (1.3)  100.50 (27.3)  2.37 (0.19)  1.20 (0.14)  395.0 (111.5)  115.0 (14.5)  2.1 (0.8)  5.73 (0.84)  3.80 (1.11)  1.20 (0.32)  1.23 (1.42)  4.45 (1.45) | 4.08 to 11.37  13.5 to 17.4  142 to 350  40.1 to 50.6  80.6 to 95.5  26.9 to 32.3  31.9 to 35.3  66 to 83  35 to 52  23 to 35  1.10 to 2.10  5 to 21  < 50  < 50  30 to 120  136 to 146  3.5 to 5.1  101 to 109  2.8 to 7.2  59 to 104  2.20 to 2.65  0.81 to 1.45  208 to 428  77 to 155  0.3 to 4.0  < 5.17 = N, 5.17 to 6.18 = BD, ≥ 6.21 = H;  < 3.4 = N;  ≥ 1.55 = E, < 1.03 = L;  < 1.7 = N;  3.9 to 5.6 | 0.043*  0.089  0.247  0.043*  0.315  0.353  0.529  0.423  0.125  0.343  0.056  0.082  0.252  0.093  0.504  0.971  0.912  0.075  0.463  0.075  0.165  0.481  0.853  0.853  0.481  0.089  0.075  0.912  0.853  0.315 |

* statistical significance at p < 0.05, IQR = interquartile range, N = normal, BD = borderline high, H = high, E = excellent level, L = lower than desirable
