# Supplementary material for: Kratom use disorder and unfolded protein response: Evaluating their relationship in a case control study
Source: PLoS One. 2023 Jun 23;18(6):e0287466. doi: 10.1371/journal.pone.0287466 (PMC10289391; doi:10.1371/journal.pone.0287466)
Supplement: S2 Table — (DOCX) [file pone.0287466.s007.docx]

**Table S2. Socio-demographic and clinical characteristics of participants**

| **Variables** | **Kratom users** | | **Control subjects** | |
| --- | --- | --- | --- | --- |
|  | **N** | **%** | **N** | **%** |
| **Age:**  < 40 years  ≥ 40 years  **Ethnicity:**  Malays  Others  **Marital status:**  Married  Single/divorce/widower  **Education status:**  Up to secondary education  Tertiary education  **Employment status:**  Employed  Non-employed/student  **Monthly income:**  ≤ RM 1000  > RM 1000  **Cigarette smoking:**  Non-smoker  ≤ 10 stick/day  > 10 sticks/day  **Mean body mass index (kg/m^2^)**  **Blood pressure:**  Normal (< 130/80 mmHg)  High (≥ 130/80 mmHg)  **Pulse rate:**  Normal (< 100 beats/minute  Tachycardia (≥ 100 beats/minute) | 17  43  58  2  50  10  47  13  58  2  2  58  0  19  41  26.19^#^  23  37  56  4 | 28.3  71.7  96.7  3.3  83.3  16.7  78.3  21.7  96.7  3.3  3.3  96.7  0.0  31.7  68.3  6.44^$^  38.3  61.7  93.3  6.7 | 46  4  44  6  30  20  5  45  41  9  16  34  43  7  0  27.41^#^  36  14  43  7 | 92.0  8.0  88.0  12.0  60.0  40.0  10.0  90.0  82.0  18.0  32.0  68.0  86.0  14.0  0.0  6.32^$^  72.0  28.0  86.0  14.0 |

^#^ = median, ^$^ = interquartile range
