# Supplementary material for: Kratom use disorder and unfolded protein response: Evaluating their relationship in a case control study
Source: PLoS One. 2023 Jun 23;18(6):e0287466. doi: 10.1371/journal.pone.0287466 (PMC10289391; doi:10.1371/journal.pone.0287466)
Supplement: S3 Table — (DOCX) [file pone.0287466.s008.docx]

**Table S3. Characteristics of kratom use among the regular kratom users**

| **Variables** | **Number of**  **participants (n)** | **Percentage**  **(%)** |
| --- | --- | --- |
| **Kratom use duration:**  1–6 years  > 6 years  **Kratom use frequency:**  1-3 times per day  > 3 times per day  **Kratom use quantity:**  1–3 glasses per day  > 3 glasses per day  **Kratom use disorder:**  No  Yes | 16  44  34  26  44  16  35  25 | 26.7  73.3  56.7  43.3  73.3  26.7  58.3  41.7 |
